# Prostate Safety Events During Testosterone Replacement Therapy in Men With Hypogonadism: A Randomized Clinical Trial

**DOI:** 10.1001/jamanetworkopen.2023.48692

**Published:** 2023-12-27

**Authors:** Shalender Bhasin, Thomas G. Travison, Karol M. Pencina, Michael O’Leary, Glenn R. Cunningham, A. Michael Lincoff, Steven E. Nissen, M. Scott Lucia, Mark A. Preston, Mohit Khera, Nader Khan, Michael C. Snabes, Xue Li, Catherine M. Tangen, Kevin A. Buhr, Ian M. Thompson

**Affiliations:** 1 Research Program in Men’s Health: Aging and Metabolism, Boston Claude D. Pepper Older Americans Independence Center, Brigham and Women’s Hospital, Harvard Medical School, Boston, Massachusetts; 2 Marcus Institute for Aging Research, Hebrew Senior Life, Division of Gerontology, Beth Israel Deaconess Medical Center, Harvard Medical School, Boston, Massachusetts; 3 Baylor College of Medicine, Houston, Texas; 4 Cleveland Clinic Coordinating Center for Clinical Research (C5Research), Department of Cardiovascular Medicine, Cleveland Clinic, Cleveland, Ohio; 5 Department of Pathology, University of Colorado, Aurora; 6 Division of Urology, Brigham and Women’s Hospital, Boston, Massachusetts; 7 AbbVie Inc, North Chicago, Illinois; 8 Fred Hutchison Cancer Center, University of Washington, Seattle; 9 Statistical Data Analysis Center, Department of Biostatistics and Medical Informatics, University of Wisconsin, Madison; 10 CHRISTUS Santa Rosa Health System and The University of Texas Health Science Center, San Antonio

## Abstract

**Importance:**

The effect of testosterone replacement therapy (TRT) on the risk of prostate cancer and other adverse prostate events is unknown.

**Objective:**

To compare the effect of TRT vs placebo on the incidences of high-grade prostate cancers (Gleason score ≥4 + 3), any prostate cancer, acute urinary retention, invasive prostate procedures, and pharmacologic treatment for lower urinary tract symptoms in men with hypogonadism.

**Design, Setting, and Participants:**

This placebo-controlled, double-blind randomized clinical trial enrolled 5246 men (aged 45-80 years) from 316 US trial sites who had 2 testosterone concentrations less than 300 ng/dL, hypogonadal symptoms, and cardiovascular disease (CVD) or increased CVD risk. Men with prostate-specific antigen (PSA) concentrations greater than 3.0 ng/mL and International Prostate Symptom Score (IPSS) greater than 19 were excluded. Enrollment took place between May 23, 2018, and February 1, 2022, and end-of-study visits were conducted between May 31, 2022, and January 19, 2023.

**Intervention:**

Participants were randomized, with stratification for prior CVD, to topical 1.62% testosterone gel or placebo.

**Main Outcomes and Measures:**

The primary prostate safety end point was the incidence of adjudicated high-grade prostate cancer. Secondary end points included incidence of any adjudicated prostate cancer, acute urinary retention, invasive prostate surgical procedure, prostate biopsy, and new pharmacologic treatment. Intervention effect was analyzed using a discrete-time proportional hazards model.

**Results:**

A total of 5204 men (mean [SD] age, 63.3 [7.9] years) were analyzed. At baseline, the mean (SD) PSA concentration was 0.92 (0.67) ng/mL, and the mean (SD) IPSS was 7.1 (5.6). The mean (SD) treatment duration as 21.8 (14.2) months in the TRT group and 21.6 (14.0) months in the placebo group. During 14 304 person-years of follow-up, the incidence of high-grade prostate cancer (5 of 2596 [0.19%] in the TRT group vs 3 of 2602 [0.12%] in the placebo group; hazard ratio, 1.62; 95% CI, 0.39-6.77; *P* = .51) did not differ significantly between groups; the incidences of any prostate cancer, acute urinary retention, invasive surgical procedures, prostate biopsy, and new pharmacologic treatment also did not differ significantly. Change in IPSS did not differ between groups. The PSA concentrations increased more in testosterone-treated than placebo-treated men.

**Conclusions and Relevance:**

In a population of middle-aged and older men with hypogonadism, carefully evaluated to exclude those at high risk of prostate cancer, the incidences of high-grade or any prostate cancer and other prostate events were low and did not differ significantly between testosterone- and placebo-treated men. The study’s findings may facilitate a more informed appraisal of the potential risks of TRT.

**Trial Registration:**

ClinicalTrials.gov Identifier: NCT03518034

## Key Points

**Question:**

Does testosterone replacement therapy in men with hypogonadism increase the risk of high-grade or any prostate cancer or other adverse prostate events?

**Findings:**

During 14 304 person-years of follow-up of 5204 men (aged 45-80 years) with hypogonadism in this randomized clinical trial, incidences of high-grade or any prostate cancer, acute urinary retention, invasive surgical procedures, and new pharmacologic treatment were low and did not differ significantly between groups.

**Meaning:**

The study’s findings will facilitate a more informed appraisal of the potential prostate risks of testosterone replacement therapy.

## Teaser

This randomized clinical trial examines the effects of testosterone replacement therapy on major adverse cardiovascular events in men with hypogonadism.

## Introduction

The relationship between testosterone replacement therapy (TRT) and the risk of prostate cancer remains incompletely studied.^1,2,3^ Epidemiologic studies have not found a consistent association between prostate cancer risk and testosterone levels or polymorphisms in genes involved in androgen action.^4,5,6,7,8,9,10,11,12,13^ Prostate events were not adjudicated in any testosterone trial, and none have reported the incidence of high-grade prostate cancer or other prostate events, such as acute urinary retention, invasive prostate procedures, or initiation of new pharmacologic therapy for benign prostatic hyperplasia (BPH).^1,14^ Because of uncertainty about the risk of prostate events during TRT, most professional society guidelines recommend against TRT in men with a history or increased risk of prostate cancer.^1,2,15^

In 2015, the US Food and Drug Administration required testosterone manufacturers to conduct a randomized clinical trial to determine the effect of TRT on major adverse cardiovascular events (MACEs).^16^ The Testosterone Replacement Therapy for Assessment of Long-Term Vascular Events and Efficacy Response in Hypogonadal Men (TRAVERSE) study was designed to meet this regulatory requirement.^17^ Because of its large size and longer duration, the TRAVERSE study offered a unique opportunity to evaluate the effects of TRT on prostate safety events.^17^ The study compared the effects of TRT and placebo on the incidences of high-grade prostate cancer, any prostate cancer, acute urinary retention, invasive prostate surgical procedures for BPH, and initiation of pharmacologic therapy for BPH. Prostate events were recorded using a structured protocol and adjudicated. To minimize ascertainment bias due to greater likelihood of urologic referral for prostate biopsy because of testosterone-induced elevation in prostate-specific antigen (PSA) concentrations, the TRAVERSE study protocol prespecified procedures for managing PSA elevations and urologic referrals.

## Methods

This randomized clinical trial’s design, as well as the MACEs and overall safety results, have been previously published.^17,18^ Briefly, this placebo-controlled, double-blind, parallel-group randomized clinical trial enrolled men, aged 45 to 80 years, with 2 fasting, morning testosterone concentrations, measured using liquid chromatography–tandem mass spectrometry, less than 300 ng/dL (to convert to nanomoles per liter, multiply by 0.0347) in a central laboratory certified by the Hormone Standardization Program for Testosterone, 1 or more symptoms of hypogonadism, and prior cardiovascular disease (CVD) or increased risk of CVD.^17^ Men with history of prostate cancer, PSA concentrations greater than 3.0 ng/mL (or >1.5 ng/mL if receiving a steroid 5α-reductase inhibitor [5ARI] [to convert to micrograms per liter, multiply by 1]), severe lower urinary tract symptoms (LUTSs) (International Prostate Symptom Score [IPSS] >19), or a prostate nodule or induration were excluded. A PSA cutoff of 3 ng/mL was established to exclude men at increased prostate cancer risk.^19^ Participants were randomized in a 1:1 ratio with stratification for preexisting CVD to receive 1.62% transdermal testosterone gel or matching placebo gel for the duration of the study. Testosterone dose was adjusted, while maintaining blinding, based on on-treatment testosterone and hematocrit levels to maintain testosterone concentrations between 350 and 750 ng/dL and hematocrit levels less than 54% (to convert to a proportion of 1.0, multiply by 0.01).^17,18^ Participants’ self-reported race and ethnicity were collected because racial differences in the incidence of clinical prostate cancers are well recognized. The trial was conducted at 316 US sites. Enrollment took place between May 23, 2018, and February 1, 2022, and end-of-study visits were conducted between May 31, 2022, and January 19, 2023. The study protocol was approved by the national and local institutional review boards for human subjects research. All participants provided written informed consent. An independent data and safety monitoring board reviewed safety data every 6 months.

### Prostate Safety Monitoring Plan

The prespecified prostate safety monitoring plan is provided in Supplement 1. The PSA levels were measured at baseline, 3 months, 12 months, and annually thereafter, and IPSS was assessed at baseline, 3 months, 12 months, 36 months, and the end of the study. Digital prostate examinations were performed at baseline, 12 months, 36 months, and end of study. At each visit, participants were asked structured questions about LUTS and prostate procedures. If a prostate procedure was reported, an attempt was made to obtain pathology reports and tissue.

To minimize the ascertainment bias attributable to the increased risk of being referred for a prostate biopsy because of testosterone-induced increase in PSA levels, the criteria for urologic referral were prespecified. The participants were referred for urologic evaluation and possible biopsy if they had any of the following: (1) confirmed PSA increase more than 1.4 ng/mL above baseline in the first year of treatment (or >0.7 ng/mL in 5ARI-treated men); (2) confirmed PSA concentration greater than 4.0 ng/mL at any time (>2.0 ng/mL in 5ARI-treated men); (3) for men aged 45 to 54 years with a baseline PSA concentration less than 1.5 ng/mL, a PSA level increasing to 3.0 ng/mL at any time (<0.75 ng/mL increasing to 1.5 ng/mL for 5ARI-treated men); or (4) prostate nodule or induration at any time. For criteria 1, 2, and 3, elevations in PSA concentrations were confirmed by repeating the test.^20,21^ For the men who met these criteria, prostate cancer risk was estimated using the Prostate Cancer Prevention Trial Risk Calculator, version 2.0 (UT Health San Antonio), and participants were provided an institutional review board–approved video that described the potential benefits and risks of prostate biopsy^22^ to facilitate informed decision-making regarding prostate biopsy.

### Prostate Safety End Points

The primary prostate safety end point was the incidence of high-grade prostate cancer (Gleason score 4 + 3 or higher). Secondary end points included the incidence of any prostate cancer, acute urinary retention, invasive prostate surgical procedure for BPH, prostate biopsy, and new pharmacologic treatment for LUTSs. The LUTSs were evaluated using the IPSS. Changes in PSA concentrations from baseline and from month 12 were determined.

### Adjudication of Prostate Safety End Points

A blinded Prostate Adjudication Committee adjudicated prostate cancer diagnosis and Gleason score, acute urinary retention, and invasive prostate surgical procedure for BPH. The diagnosis of prostate cancer was based on evaluation of tissue from prostate biopsy specimens and surgical procedures by the Prostate Adjudication Center at the University of Colorado. If tissue or slides were not available, the Prostate Adjudication Committee reviewed site pathology reports. High-grade prostate cancer was defined as a Gleason score of 4 + 3 or higher.^23^ Acute urinary retention was defined as inability to voluntarily pass urine, requiring a visit to the emergency department, and/or placement of a catheter, ascertained by participant self-report and verified by medical record. An invasive prostate procedure was defined as any surgical procedure for BPH other than a prostate biopsy, verified by medical record.

### Statistical Analysis

The trial’s statistical analysis plan is available in Supplement 2. Analyses used SAS software, version 9.4 (SAS Institute Inc) and R, version 4.2.1 (R Foundation for Statistical Computing).^24^ Descriptive analyses of baseline characteristics were conducted in the full analysis set, which included all randomized participants. Prostate safety analyses were conducted in the safety set, which included all randomized participants who received at least 1 dose of the study drug. The data analysis and interpretation of the data were performed by the statisticians associated with the Prostate Substudy Committee (K.B., K.M.P., and T.G.T.).

Analysis of the primary safety end point and event-based secondary end points used a discrete-time proportional hazards model^25^ with event intervals based on scheduled visits. All postrandomization events were included. Hazard ratios (HRs) for treatment effect and associated 95% CIs and Wald *P* values were calculated, adjusting for prior CVD. The discrete-time model was prespecified under the assumption that exact event times might not be consistently available for analysis during the COVID-19 pandemic, a concern that proved unfounded, so an additional post hoc Cox proportional hazards analysis using actual time of events was conducted. Aalen-Johansen estimates of cumulative incidence of prostate events with death as a competing risk were calculated. Post hoc sensitivity analyses of events occurring within 1 year and within 30 days of the last dose of the study drug were also conducted.

Changes over time in IPSSs, PSA levels, and hormone levels were analyzed using linear mixed-effects models with fixed effects for treatment, visit, treatment × visit interaction, baseline value, CVD status, and a random-subject effect using an unstructured covariance. Least-squares means estimates, 95% CIs, and *P* values for treatment effect were computed using an *F* test. For PSA, a mixed model was used to test whether treatment difference continued to increase after month 12 by comparing month 12 with the mean of later visits. All hypothesis tests used a 2-sided significance level of *P* < .05.

The study was powered to establish noninferiority for the MACE end point within a noninferiority margin of an upper confidence limit of the HR less than 1.5. Approximately 6000 individuals were to be recruited to accrue 256 MACEs (90% power) under the initial assumptions of annual event rate, accrual rate, and discontinuation rate.^17^

## Results

**Figure 1.**
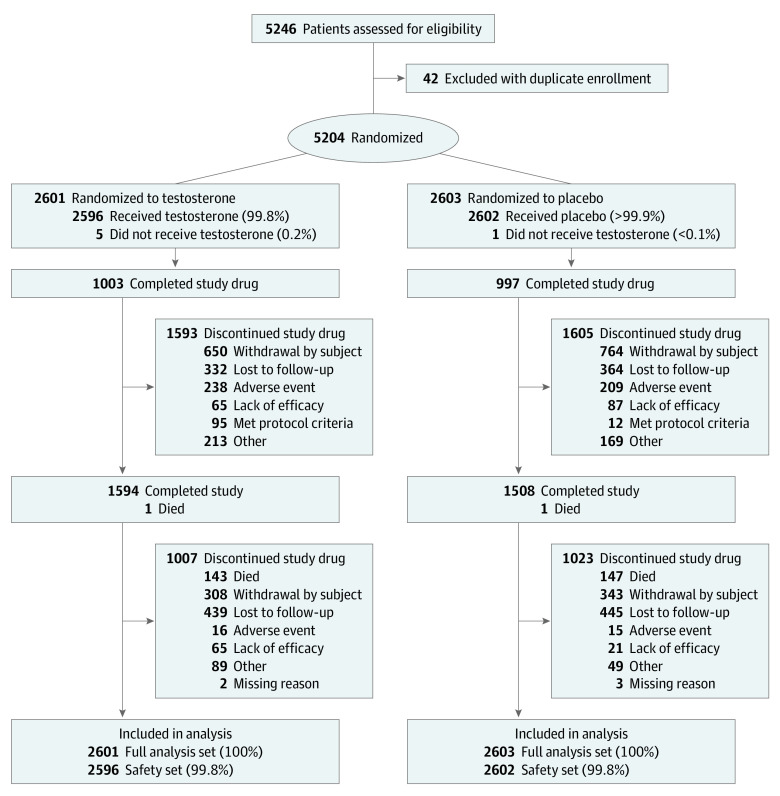
CONSORT Flow Diagram

Among 32 152 screened men, 50 (0.16%) were excluded because of a history of prostate or breast cancer, 1201 (3.74%) for PSA concentrations greater than 3.0 ng/mL (or >1.5 ng/mL if receiving 5ARIs), 549 (1.71%) for IPSSs greater than 19, and 57 (0.18%) for prostate nodule or induration; these percentages should be interpreted with caution because men who failed screening at earlier screening visits did not complete subsequent screening assessments. Among 5246 identification numbers of randomized men, 42 were attributed to 20 participants with duplicate enrollment. After excluding these duplicates, the full analysis set included 5204 participants (mean [SD] age, 63.3 [7.9] years; self-reported race: 877 [16.9%] Black, 4154 [79.8%] White, and 173 [3.3%] other; self-reported Hispanic or Latinx ethnicity, 848 [16.3%]), with 2601 in the TRT group and 2603 in the placebo group. The safety set included 5198 participants (2596 in the TRT group and 2602 in the placebo group) who received at least 1 dose of study medication (Figure 1).

As reported earlier,^18^ the mean (SD) follow-up duration was 33.0 (12.1) months. Of 5204 participants in the full analysis set, 4804 (92.3%) were followed up for at least 1 one year, 3842 (73.9%) for 2 years, 2974 (57.2%) for 3 years, and 85 (1.6%) for 4 years, yielding 14 304 person-years of follow-up. The mean (SD) treatment duration was 21.8 (14.2) months in the TRT group and 21.6 (14.0) months in the placebo group, and treatment discontinuation rates were similar between the 2 arms.

Baseline characteristics of the participants have been previously published.^18^ The mean (SD) PSA concentration was 0.92 (0.67) ng/mL. Of 5182 men with nonmissing baseline PSA values, 3347 (64.6%) had PSA concentrations less than 1 ng/mL, 1355 (26.1%) had PSA concentrations between 1.00 and 1.99 ng/mL, and 480 (9.3%) had PSA concentrations between 2 and 3 ng/mL. The mean (SD) baseline IPSS was 7.1 (5.6).

### High-Grade and All Prostate Cancers

**Figure 2.**
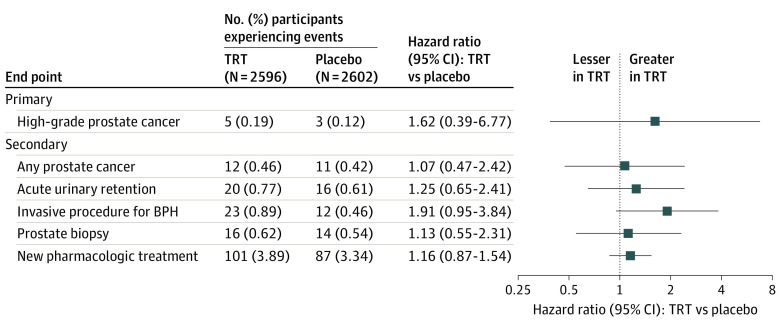
Incidence of Primary (High-Grade Prostate Cancer) and Secondary Prostate Safety End Points Discrete-time proportional hazards model estimates of hazard ratios (95% CIs) quantifying differential risk in testosterone replacement therapy (TRT) relative to placebo are shown in the forest plot. The hazard ratios are the hazard in the TRT group over the hazard in the placebo group, so a value greater than 1 indicates an excess of prostate events in the TRT group. BPH indicates benign prostate hyperplasia.

**Figure 3.**
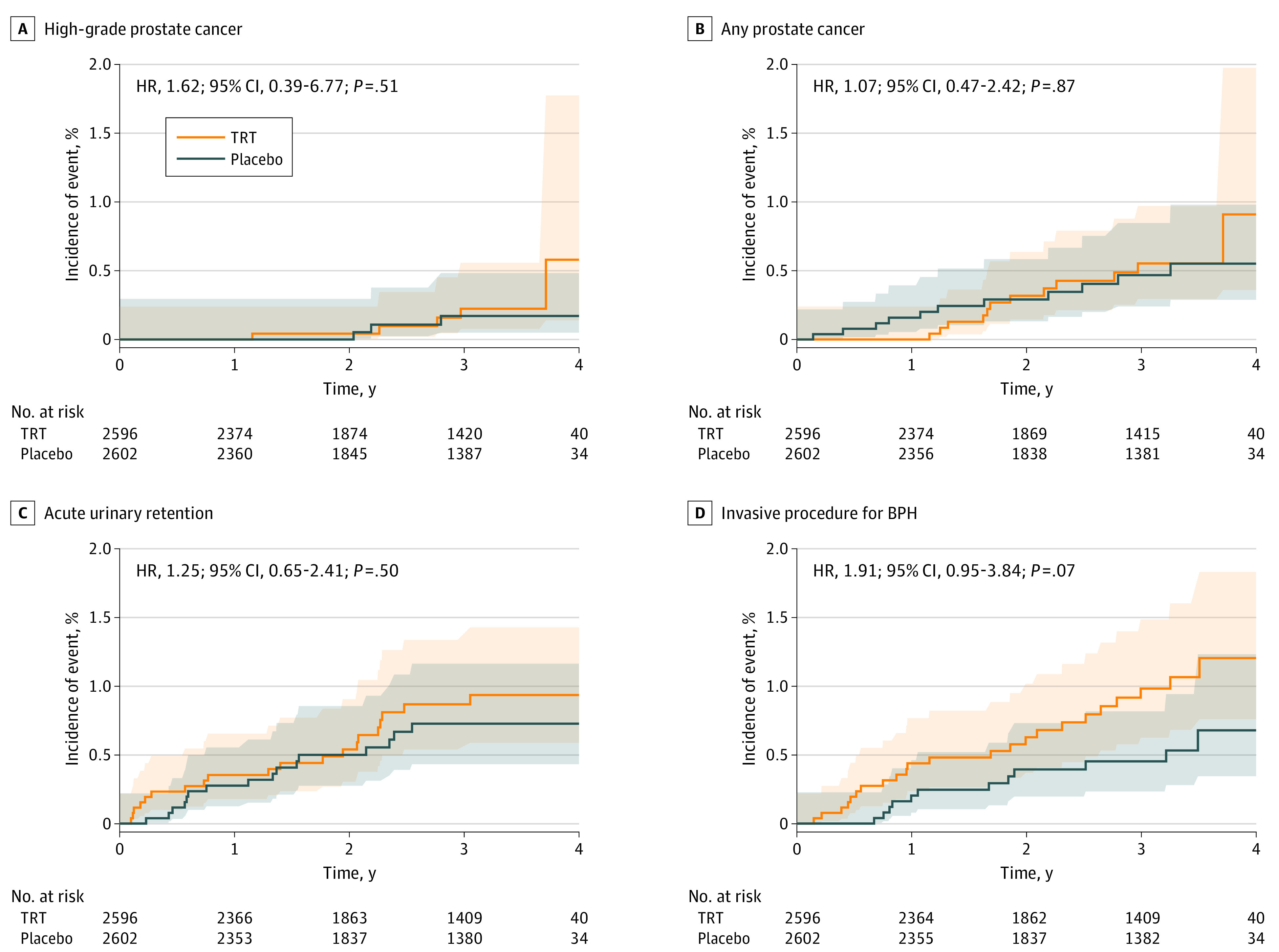
Estimated Cumulative Incidences of Primary and Secondary Event-Based Outcomes as a Function of Time From Baseline Aalen-Johansen estimates of cumulative incidence with death as a competing risk, together with pointwise 95% CIs, are shown. Hazard ratios (HRs) and associated 95% CIs and *P* values based on the discrete proportional hazards model are also shown. Between-group differences are not statistically significant. BPH indicates benign prostatic hyperplasia; HR, hazard ratio; and TRT, testosterone replacement therapy.

As reported previously in the trial’s overall safety events,^18^ during 14 304 person-years of follow-up, there were 5 participants with high-grade prostate cancer in the TRT group and 3 in the placebo group. The incidence of high-grade prostate cancer did not differ significantly between groups (5 of 2596 [0.19%] in the TRT group vs 3 of 2602 [0.12%] in the placebo group; hazard ratio, 1.62; 95% CI, 0.39-6.77; *P* = .51) (Figure 2 and Figure 3). Among the 8 participants with high-grade cancer, 3 had baseline PSA concentrations between 1 and 1.99 ng/mL and 5 between 2 and 3 ng/mL.

The number of participants with any prostate cancer did not differ between the TRT (12 [0.46%]) and placebo (11 [0.42%]) groups (HR, 1.07; 95% CI, 0.47-2.42; *P* = .87). Among 23 men with prostate cancer, 1 had a baseline PSA concentration less than 1 ng/mL, 8 between 1 and 1.99 ng/mL, and 14 between 2 and 3 ng/mL. The highest postbaseline PSA concentration before biopsy in these 23 men is shown in eTable 1 in Supplement 3.

### Other Secondary Prostate Safety End Points

Twenty testosterone-treated men (0.77%) and 16 placebo-treated men (0.61%) developed acute urinary retention, with no significant difference between groups (HR, 1.25; 95% CI, 0.65-2.41; *P* = .50). Twenty-three men (0.89%) in the TRT group underwent an invasive surgical prostate procedure compared with 12 (0.46%) in the placebo group (HR, 1.91; 95% CI, 0.95-3.84; *P* = .07). Rates of new pharmacologic therapy for LUTSs did not differ significantly between the TRT and placebo groups (101 [3.89%] vs 87 [3.34%]; HR, 1.16; 95% CI, 0.87-1.54; *P* = .32) (Figure 2 and Figure 3).

Eighty-five men (1.6%) met the criteria for referral for urologic evaluation, 57 (2.2%) in the TRT group vs 28 (1.1%) in the placebo group. Sixty men (39 in the TRT group and 21 in the placebo group) had confirmed PSA concentrations greater than 4.0 ng/mL, 37 men (25 in the TRT group and 12 in the placebo group) had confirmed increases in PSA concentrations greater than 1.4 ng/mL above baseline during the first year (or >0.7 ng/mL for those taking 5ARIs), 5 men (4 in the TRT group and 1 in the placebo group) had a new prostate nodule or induration, and 1 man (in the TRT group) had a PSA concentration that increased from less than 1.5 ng/mL at baseline to greater than 3.0 ng/mL.

Of the 85 men who met the criteria for urologic referral, 16 (18.8%) elected to undergo prostate biopsy; an additional 14 men who did not meet these criteria also underwent biopsy. The numbers of prostate biopsies (16 in the TRT group vs 14 in the placebo group) did not differ between groups (0.62% vs 0.54%; HR, 1.13; 95% CI, 0.55-2.31; *P* = .74). Eighteen men who underwent biopsy had baseline PSA concentrations between 2 and 3 ng/mL.

### Post Hoc Sensitivity Analyses

Post hoc analysis for primary and secondary event end points using a Cox proportional hazards regression model (eFigure 1 in Supplement 3) yielded results similar to those of prespecified analyses. Similarly, the results of the sensitivity analyses in which events 1 year and 30 days after the end of treatment were censored (eFigures 2 and 3 in Supplement 3) were similar to those of prespecified analyses.

### Lower Urinary Tract Symptoms

**Figure 4.**
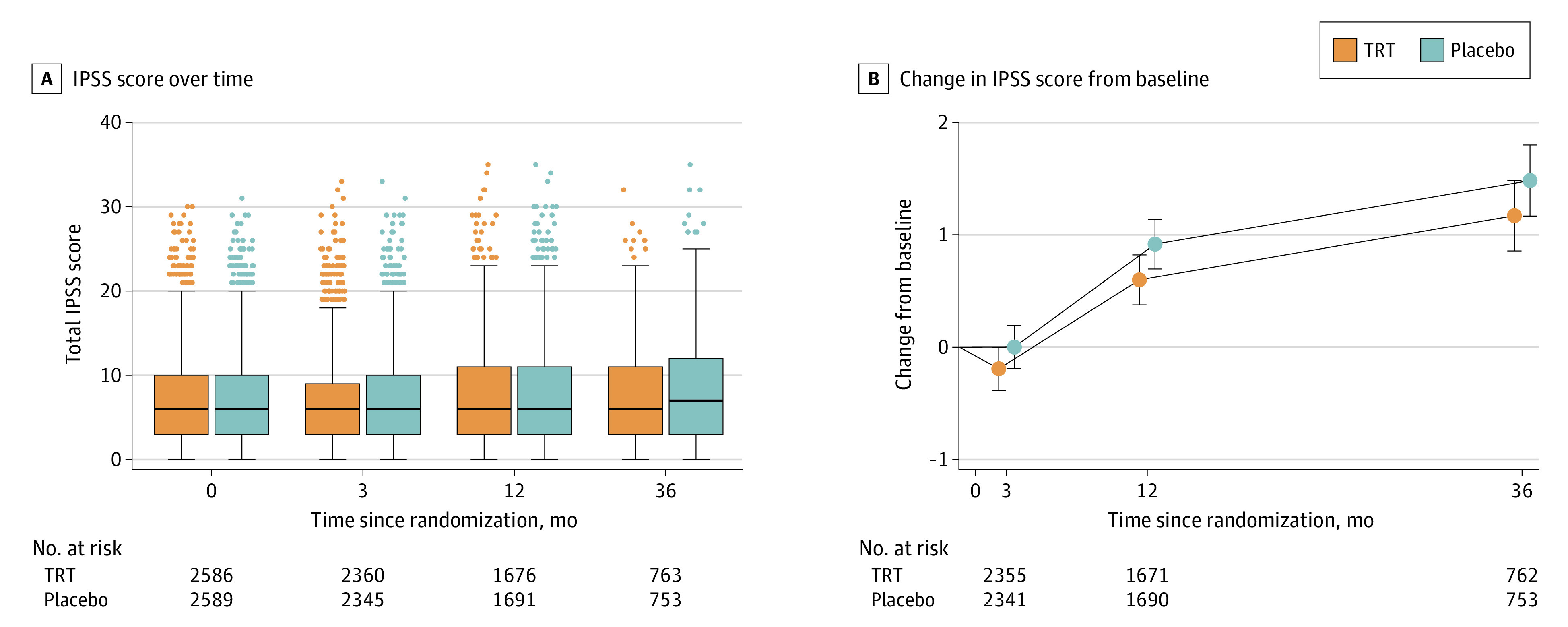
Changes in Lower Urinary Tract Symptoms Over Time The lower urinary tract symptoms were evaluated using the International Prostate Symptom Score (IPSS). TRT indicates testosterone replacement therapy.

The IPSS increased over time in both groups (Figure 4); change from baseline in IPSS did not differ significantly between groups. Of 4809 men with any postbaseline IPSS, 378 (7.9%) had a score greater than 19 (180 [7.5%] in the TRT group and 198 [8.2%] in the placebo group).

### PSA Levels

Testosterone treatment was associated with a greater increase in PSA levels than placebo (estimated between-group difference, 0.11 [ 95% CI, 0.07-0.15] ng/mL at 3 months; 0.15 [95% CI, 0.08-0.21] ng/mL at 12 months; 0.11 [95% CI, −0.01 to 0.21] ng/mL at 24 months; 0.01 [95% CI, −0.09 to 0.10] ng/mL at 36 months; and 0.09 [95% CI, −0.04 to 0.22] ng/mL at 48 months; omnibus test *P* < .001) (eFigure 4 in Supplement 3) regardless of baseline PSA concentration (Table). There was no significant between-group difference in PSA levels after month 12; the difference at time points after month 12 was significantly smaller than difference at month 12.

**Table.** Change From Baseline in Serum PSA Levels in Study Participants Categorized by Baseline PSA Levels

Treatment	No.	PSA level, mean (SD)	Change from baseline, least-squares mean (95% CI)	Treatment difference, least-squares mean (95% CI)
**Baseline PSA <1 ng/mL**				
Month 0				
TRT	1572	0.51 (0.24)	NA	NA
Placebo	1549	0.51 (0.24)	NA	NA
Month 3				
TRT	1557	0.65 (0.48)	0.14 (0.10 to 0.18)	0.07 (0.01 to 0.12)
Placebo	1529	0.59 (0.63)	0.07 (0.03 to 0.11)	NA
Month 12				
TRT	1122	0.71 (0.68)	0.19 (0.15 to 0.24)	0.06 (−0.01 to 0.12)
Placebo	1075	0.65 (1.42)	0.14 (0.09 to 0.19)	NA
Month 24				
TRT	762	0.72 (0.84)	0.20 (0.15 to 0.26)	0.13 (0.05 to 0.21)
Placebo	696	0.59 (0.48)	0.08 (0.02 to 0.13)	NA
Month 36				
TRT	446	0.72 (0.67)	0.20 (0.12 to 0.27)	−0.02 (−0.12 to 0.08)
Placebo	432	0.74 (1.26)	0.22 (0.15 to 0.29)	NA
Month 48				
TRT	128	0.74 (0.49)	0.21 (0.08 to 0.34)	−0.03 (−0.22 to 0.15)
Placebo	119	0.83 (1.64)	0.24 (0.11 to 0.38)	NA
**Baseline PSA 1 to <2 ng/mL**				
Month 0				
TRT	642	1.40 (0.28)	NA	NA
Placebo	630	1.39 (0.28)	NA	NA
Month 3				
TRT	635	1.64 (0.82)	0.24 (0.17 to 0.30)	0.18 (0.09 to 0.28)
Placebo	624	1.44 (0.96)	0.05 (−0.01 to 0.12)	NA
Month 12				
TRT	454	1.65 (0.87)	0.26 (0.18 to 0.33)	0.26 (0.16 to 0.37)
Placebo	478	1.38 (0.64)	−0.01 (−0.08 to 0.07)	NA
Month 24				
TRT	338	1.60 (0.93)	0.20 (0.11 to 0.28)	0.18 (0.06 to 0.30)
Placebo	307	1.38 (0.76)	0.02 (−0.07 to 0.11)	NA
Month 36				
TRT	230	1.68 (0.95)	0.27 (0.17 to 0.37)	0.10 (−0.05 to 0.24)
Placebo	213	1.51 (1.14)	0.17 (0.07 to 0.28)	NA
Month 48				
TRT	74	1.75 (0.85)	0.36 (0.19 to 0.53)	0.10 (−0.16 to 0.37)
Placebo	51	1.68 (0.90)	0.25 (0.05 to 0.45)	NA
**Baseline PSA 2-3 ng/mL**				
Month 0				
TRT	195	2.46 (0.28)	NA	NA
Placebo	251	2.43 (0.30)	NA	NA
Month 4				
TRT	192	2.74 (1.09)	0.29 (−0.02 to 0.60)	0.20 (−0.21 to 0.62)
Placebo	250	2.52 (1.95)	0.08 (−0.19 to 0.36)	NA
Month 12				
TRT	146	2.91 (1.22)	0.46 (0.10 to 0.81)	0.45 (−0.03 to 0.93)
Placebo	176	2.43 (1.33)	0.01 (−0.32 to 0.33)	NA
Month 24				
TRT	93	2.97 (3.29)	0.59 (0.15 to 1.03)	0.06 (−0.53 to 0.66)
Placebo	112	2.90 (4.91)	0.53 (0.13 to 0.93)	NA
Month 36				
TRT	60	2.74 (1.03)	0.29 (−0.25 to 0.83)	0.21 (−0.52 to 0.94)
Placebo	72	2.41 (1.05)	0.08 (−0.41 to 0.57)	NA
Month 48				
TRT	17	3.11 (1.44)	0.52 (−0.48 to 1.52)	0.51 (−0.78 to 1.80)
Placebo	25	2.24 (0.97)	0.01 (−0.81 to 0.83)	NA

Abbreviations: NA, not applicable; PSA, prostate-specific antigen; TRT, testosterone replacement therapy.

SI conversion factor: To convert PSA to micrograms per liter, multiply by 1.

### Hormone Levels

Mean (SD) total testosterone was 220 (48) ng/dL at baseline.^18^ As reported, testosterone and estradiol levels,^18^ as well as dihydrotestosterone levels (eTable 2 in Supplement 3), increased significantly in testosterone-treated men but did not change in placebo-treated men.

## Discussion

The TRAVERSE study is, to our knowledge, the largest randomized trial of TRT conducted to date, with prospectively recorded and adjudicated prostate safety outcomes. Among middle-aged and older men with hypogonadism who had or were at increased risk of CVD, the incidence of high-grade or any prostate cancer in TRT-treated men with a baseline PSA concentration less than 3.0 ng/mL was low and not significantly different from that in placebo-treated men. This group of men whose PSA concentration is less than 3.0 ng/mL represents most of the aging US population.^26^ Similarly, incidences of acute urinary retention, invasive surgical procedure for BPH, or new pharmacologic treatment for LUTSs did not differ between the treatment groups. The invasive prostate surgical procedures were more common in the TRT group compared with the placebo group, although the difference was not significant. Consistent with meta-analyses of smaller testosterone trials, TRT did not increase IPSSs.^14,27^ Although PSA concentrations increased more among the TRT group than the placebo group, the mean increase was small and between-group difference did not widen after 12 months. Thus, in a population men with hypogonadism and PSA concentrations less than 3 ng/mL who were evaluated carefully to exclude those at increased prostate cancer risk, TRT was associated with low risk of adverse prostate events, including cancer.

Prostate cancer is highly prevalent among older men, but only a small fraction have high-grade tumors.^19^ Androgen receptor signaling plays a central role in prostate cancer biology, and testosterone treatment promotes the growth of metastatic prostate cancer.^28^ A mendelian randomization analysis found an increased incidence of prostate cancer in men with higher genetically determined testosterone level^29^; conversely, men with Klinefelter syndrome have lower risk of prostate cancer.^30^ These data have led to concerns that TRT could promote progression of subclinical low-grade prostate cancer.^1^ Because TRT increases PSA in men with hypogonadism, PSA elevations in older men receiving TRT could lead to prostate biopsy and detection of a subclinical low-grade prostate cancer.^1^ To minimize the risk of unnecessary prostate biopsies and mitigate ascertainment bias, while enabling detection of prostate cancers for which clinical management may reduce long-term disease-related morbidity and mortality, the study protocol specified PSA elevation thresholds for referral to a urologist.^21,31^ Elevations in PSA concentrations above these thresholds were verified, and participants with confirmed PSA elevation were asked to watch a video on the significance of PSA elevation and the benefits and risks of prostate biopsy to facilitate a shared decision on prostate biopsy. This approach was effective in reducing the number of prostate biopsies in both treatment groups; the small number of biopsies and high percentage of positive biopsy results in the trial support its usefulness in facilitating shared decision-making before prostate biopsy in men receiving TRT.

### Limitations

The trial has some limitations. These findings should not be applied to patients with known prostate cancer, those with higher PSA values, or men who do not have confirmed hypogonadism. Although the TRAVERSE study was longer than most other randomized clinical trials of TRT, carcinogens may require many years to induce malignant neoplasms. The trial’s structured evaluation of men after PSA testing did not include prostate imaging or other biomarker tests that may influence the decision to perform a biopsy. It is possible that shared decision-making played a role in lower rates of prostate biopsy; results could be different in a setting in which shared decision-making is not made available. Although the trial’s sample size is the largest of any randomized testosterone trials to date, the numbers and incidences of any prostate cancer and high-grade prostate cancer were low. Because of the small number of prostate cancer events, these findings should not be interpreted to imply that the risk of prostate cancer in the testosterone and placebo groups was similar. The trial’s findings indicate that in men with hypogonadism who were screened and monitored carefully using a structured protocol, the risk of high-grade or any prostate cancer and other prostate events is low. The trial’s findings do not apply to men at high risk of prostate cancer, who were excluded. Rates of study medication discontinuation and loss to follow-up were high, although not dissimilar from those in randomized trials in other symptomatic conditions^32,33^ or in hypogonadal men prescribed TRT.^34^ The trial was conducted during the COVID-19 pandemic, which affected retention. However, nonretention rates were similar in the 2 groups. Among participants who discontinued trial participation, nearly half did so after end-of-study visits had started, and findings were similar in sensitivity analyses limited to follow-up durations of 1 month or 1 year after the last administered dose. The study population met the Endocrine Society’s criteria for hypogonadism^1^ but had high rates of diabetes, obesity, and other comorbid conditions, not dissimilar from men with hypogonadism^35^ receiving TRT in the US.^36^

## Conclusions

In this randomized clinical trial of men with hypogonadism who were carefully evaluated to exclude those at high risk for prostate cancer and followed using a standardized monitoring plan, TRT was associated with low and similar incidences of high grade or any prostate cancer, acute urinary retention, and invasive surgical procedures for BPH compared with a placebo. Testosterone replacement therapy did not worsen LUTSs. The concern about prostate risk heavily influences decision-making by clinicians and patients who are considering TRT for hypogonadism. The study’s findings will facilitate a more informed appraisal of the potential risks of TRT.

## Notes

Supplement 1.
TRAVERSE Trial Prostate Safety Monitoring Plan

Supplement 2.
Statistical Analysis Plan

Supplement 3. **eAppendix 1. **TRAVERSE Trial Sites and Investigators **eAppendix 2.** The TRAVERSE Prostate Study Committee **eFigure 1.** Sensitivity Analysis of the Primary and Major Secondary Prostate Safety Endpoints Using a Cox Proportional Hazards Model **eFigure 2. **Sensitivity Analysis of the Primary and Major Secondary Prostate Safety Endpoints Using the Same Discrete Proportional Hazards Model as the Primary Analysis but Censoring Events Occurring After One Year Following End of Treatment **eFigure 3. **Sensitivity Analysis of the Primary and Major Secondary Prostate Safety Endpoints Using the Same Discrete Proportional Hazards Model as the Primary Analysis but Censoring Events Occurring After 30 Days Following End of Treatment **eFigure 4. **Changes in PSA Levels From Baseline in the TRT and Placebo Groups **eTable 1.** Maximum Attained PSA Values in ng/mL Prior to Prostate Biopsy for Men Diagnosed With Prostate Cancer **eTable 2.** Serum Dihydrotestosterone Concentrations

Supplement 4.
Data Sharing Statement
