# Supplementary material for: Prostate Safety Events During Testosterone Replacement Therapy in Men With Hypogonadism: A Randomized Clinical Trial
Source: JAMA Netw Open. 2023 Dec 27;6(12):e2348692. doi: 10.1001/jamanetworkopen.2023.48692 (PMC10753401; doi:10.1001/jamanetworkopen.2023.48692)

SUPPLEMENT 2

THE TRAVERSE TRIAL: PROSTATE SAFETY MONITORING PLAN

1. OBJECTIVES

The objectives of the prostate safety monitoring protocol are to establish **eligibility criteria** that will enable the investigators to exclude individuals who have prostate cancer; those at high risk for developing prostate cancer during the intervention period; and those at high risk of being referred for prostate biopsy during the trial, without excessively limiting enrollment. The prostate monitoring protocol specifies **a standardized prostate safety monitoring plan**, which will be implemented uniformly across trial sites to carefully watch for any evidence indicating a higher risk of high grade prostate cancer in men randomized to the testosterone arm compared to those randomized to the placebo arm, while also considering potential ascertainment bias. The monitoring plan aims to minimize the risk of unnecessary prostate biopsies[1] and to mitigate ascertainment biases, while enabling detection of prostate cancers for which clinical management (e.g., surveillance and/or treatment) may reduce long-term morbidity and mortality related to the disease. The prostate **safety monitoring protocol** also describes the plan for the **ascertainment and adjudication** of prostate events.

The protocol provides an overview of the pre-specified **statistical analysis plan.** This acknowledges the potential for bias in ascertainment of a greater number of prostate events in men assigned to testosterone arm of the trial due to the sensitivity of PSA response to testosterone therapy. It therefore accounts for the likelihood of encountering a greater number of prostate biopsies in the testosterone arm than in the placebo arm of the trial.

2. SPECIFIC AIMS AND HYPOTHESIS

Primary Aim

The primary aim is to determine if testosterone replacement therapy in middle-aged and older hypogonadal men increases the risk of developing Gleason 4+3 **high grade prostate cancers** when compared with placebo treatment.

Secondary Aims

- To determine if testosterone replacement therapy increases the risk of developing any prostate cancer – high grade (Gleason 7+) or low grade - when compared with placebo treatment.
- To determine if testosterone replacement therapy increases the risk of acute urinary retention or need for surgical treatment of lower urinary tract symptoms when compared with placebo treatment
- To determine if testosterone replacement therapy worsens lower urinary tract symptoms when compared with placebo treatment.

Hypotheses:

Primary

- The likelihood of undergoing prostate biopsy will be higher in testosterone than placebo, due to PSA response to testosterone therapy that is unrelated to the presence of high-grade cancer (Gleason 4+3 or higher).
- The likelihood of diagnosis of incident high-grade prostate cancer will be similar in testosterone and placebo.
- Conversely, diagnosis of low-grade cancer may be more frequent, in absolute terms, in testosterone than in placebo, owing specifically to testosterone-induced increase in PSA levels and resulting increase in rate of biopsy in men randomized to the testosterone arm.

[1] An unnecessary prostate biopsy is defined as either (1) a biopsy that reveals no evidence of cancer or (2) a biopsy that reveals a low-grade, low-volume tumor for which diagnosis and treatment is unlikely to improve a patient's long-term health and the diagnosis of which may lead to unnecessary, potentially-morbid interventions

Secondary

- There will be no significant difference in the rates of acute urinary retention or the proportion of men undergoing surgical procedures for prostate obstruction between the two intervention arms.
- The mean change in lower urinary tract symptoms assessed using the International Prostate Symptom Score (IPSS) score will not differ between the two intervention arms.
- The mean increase in prostate-specific antigen (PSA) levels will be greater in testosterone-treated men than in placebo-treated men.

3. BACKGROUND AND RATIONALE

The relationship between testosterone administration and the risk of prostate cancer remains poorly understood (1-3). No previous randomized testosterone trial has included a sufficiently large sample of men to have adequate statistical power to determine whether testosterone administration increases the risk of prostate cancer (1). Furthermore, none of the trials has been of sufficiently long duration. Therefore, we do not know whether testosterone replacement therapy increases the risk of prostate cancer.

There is no strong evidence for the association between prostate cancer risk and circulating levels of hormones or polymorphisms in genes that encode for proteins involved in steroid hormone action or metabolism (1). For instance, an analysis of prospective epidemiologic studies found no significant association between testosterone levels and the risk of prostate cancer, although there are some inconsistencies among studies (5-21). However, androgen receptor signaling plays a central role in the biology of prostate cancer, and testosterone administration promotes the growth of metastatic prostate cancer (4). The Endocrine Society recommends against testosterone supplementation in men with prostate cancer and advocates individualized consideration of prostate cancer risk prior to treatment initiation (2); other guidelines for testosterone replacement therapy are in agreement with this recommendation (3).

There are additional complexities that should be considered in developing a prostate monitoring plan in testosterone trials. Some middle-aged and older men with low testosterone levels may have prostate cancer that has not been diagnosed. Furthermore, many middle-aged and older men harbor small foci of subclinical cancer in their prostates; we do not know whether testosterone replacement therapy might cause these small subclinical foci of cancer to grow and become clinically overt. In the Prostate Cancer Prevention Trial (PCPT), prostate cancer in the placebo-treated men was diagnosed at the end of the trial by prostate biopsy in 6.6 percent of men with a PSA level of 0.5 ng/mL or less, and it increased to 26.9 percent among those with PSA values of 3.1 to 4.0 ng/mL (22). The prevalence of high-grade cancers increased from 12.5 percent of cancers associated with PSA levels of 0.5 ng/mL or less to 25.0 percent of cancers associated with PSA levels of 3.1 to 4.0 ng/mL (22). In some uncontrolled studies, low testosterone levels have been associated with high grade prostate cancers (23-25); however, other studies have reported low testosterone levels are associated with a lower prevalence of prostate cancer (26). Although most population-based studies have not associated total or free testosterone levels with increased cancer risk (5), aggressive prostate cancers have been reported to be associated with higher levels of total and free testosterone in the Baltimore Longitudinal Study of Aging (27). In the Prostate Cancer Prevention Trial, no significant associations of total or free testosterone and risk of total, low (Gleason <7) or high-grade (Gleason 7–10) prostate cancer were observed (22).

An important objective of the screening process for the trial is to identify and exclude men who have a history of prostate cancer or are at high risk for developing a prostate cancer. Testosterone therapy increases the risk of detection of subclinical prostate disease because of increased surveillance and because of testosterone-induced increase in prostate specific antigen (PSA) levels, which may lead to increased risk of prostate biopsy (28-30). Because of the high prevalence of subclinical prostate cancer in older men, an increased number of prostate biopsies in men receiving testosterone therapy would lead to the detection of a greater number of subclinical prostate cancers in men randomized to testosterone arm than in those randomized to the placebo arm of the trial. In a meta-analysis of randomized studies, a greater proportion of men randomized to testosterone were referred for prostate biopsies, had intervention-phase PSA exceeding 4 ng/ml, or had prostate cancer diagnoses than those assigned to placebo arms; ascertainment bias was likely responsible for these differences; the older men receiving testosterone had 1.8-times the odds of experiencing a prostate related adverse event in comparison to men receiving placebo likely because of this ascertainment bias (28). It is possible and even likely that a greater number of prostate cancers will

be detected in the testosterone-treated men than in placebo-treated men in this trial due to this ascertainment bias towards greater number of prostate biopsies in men assigned to testosterone arm of the trial than in the placebo arm. Therefore, it is critically important to establish a standardized monitoring process and pre-specified criteria for referring a participant for consideration of prostate biopsy during the course of the trial and to minimize the risk of unnecessary prostate biopsies.

Because of the long intervention duration of the TRAVERSE trial, it is possible that some men may develop a new prostate cancer or other prostate events unrelated to the intervention. It is also possible that testosterone administration may cause some subclinical prostate cancers, which may have been present before but which were previously undetected, to grow during testosterone administration during the trial and become clinically overt although we do not have data to support this speculation. The monitoring process should enable identification of men who may develop adverse prostate events during the intervention period regardless of their relation to testosterone administration.

Testosterone replacement therapy increases PSA levels in hypogonadal men. The typical increase in PSA levels in healthy hypogonadal men is 0.2 to 0.3 ng/mL in young and 0.2 to 0.4 ng/mL in older men. Increments >1.4 ng/mL above baseline are unusual in any 3 to 6-month period in older men without prostate cancer, and, if confirmed, warrant urologic evaluation (28).

4. ELIGIBILITY CRITERIA FOR THE TRAVERSE TRIAL

Inclusion Criteria

1. Baseline screening PSA equal to or less than 3 ng/mL timeframe prior to registration?
2. In men using steroid 5 alpha reductase inhibitors, screening PSA level equal to or less than 1.5 ng/mL. (need to have been on the inhibitor for 3 or more months to stabilize the PSA reduction and be able to adequately evaluate?)
3. Digital rectal exam not suspicious for prostate cancer within six months prior to registration.

Exclusion Criteria

1. A diagnosis of prostate cancer, current or in the past
2. Severe lower urinary tract symptoms, as indicated by IPSS score >19
3. Prostate nodule or induration detected on digital prostate examination unless it has been evaluated and determined not be prostate cancer

Rationale for the eligibility criteria

These eligibility criteria were designed to recruit middle-aged and older men who are not at high risk of currently harboring or developing prostate cancer over the course of the trial or of being referred for a prostate biopsy during the intervention period. We will exclude men who have been diagnosed with prostate cancer or who have a prostate condition that may get worse during testosterone treatment.

The PSA screening cut-off values, listed above, were based on the consideration that the higher the PSA cutoff at entry, the greater the number of participants who will exceed the threshold for urological referral. This will increase the number of men who may be referred for prostate biopsy or who are diagnosed with prostate cancer, especially in men randomized to the testosterone group. On the other hand, lower PSA screening thresholds would reduce recruitment yield.

The PSA cutoff of 3 ng/mL was established to exclude men at increased risk of prostate cancer. A prostate nodule or induration or a PSA greater than 3.0 ng/ml may indicate a previously unrecognized prostate cancer. This cutoff will also reduce the likelihood of prostate biopsy due to a rise in PSA to >4 ng/mL due to age-related changes and test-re-test variability.

In the T Trials, 78% of men with testosterone level less than 275 ng/dl had a PSA ≤ 3.0 ng/dl. If we use a PSA cutoff of ≤ 3.0 ng/mL for everyone who meets other entry criteria for the TRAVERSE trial, we would decrease recruitment of non-Blacks by 8% when compared with a PSA cut-off of ≤4.0 or by 4% when compared with a PSA cut-off of ≤ 3.5 ng/mL. However, we chose PSA ≤ 3 as the screening cut-off in spite of this modest reduction in recruitment yield because this will lead to fewer referrals for prostate biopsy and will result in fewer biopsies.

The logistical challenges of performing digital prostate examinations during the course of a large multicenter trial at nearly 330 sites with cardiovascular expertise (?) were recognized. However, we agreed to include a screening digital rectal examination after the candidate has qualified with low testosterone level and an acceptable PSA because this is the standard of care and recommended by clinical practice guidelines. A prostate examination by the participant's primary care provider or private urologist would be acceptable provided a source document establishing the date of the examination and its findings is available. The men, who are found to have a nodule or induration may be enrolled if their urological evaluation or prostate biopsies are negative for prostate cancer.

5. PROSTATE OUTCOMES

Primary Outcome

Our primary outcome is the diagnosis of high grade prostate cancer, defined as Gleason 4+3 or higher, during the trial duration.

Secondary Outcomes

- The diagnosis of any prostate cancer
- Prostatic biopsy
- Acute urinary retention
- An invasive prostate surgical procedure (prostatectomy, transurethral prostate resection, or other prostate surgical procedure) for benign prostatic hyperplasia
- Initiation of pharmacological therapy for lower urinary tract symptoms (steroid 5 alpha reductase inhibitors or alpha adrenergic blockers)
- Change in lower urinary tract symptoms, as ascertained using the International Prostate Symptom Score (IPSS)
- Change from baseline PSA levels, change from year 1 PSA levels

Rationale for the Selection of the High Grade Prostate Cancer as the Primary Outcome

The majority of prostate cancers are diagnosed via prostate biopsy; of prostate biopsies currently performed in the United States, the majority are prompted by prostate specific antigen (PSA) levels suspicious for prostate cancer. As testosterone administration to aging men will result in a spectrum of increases in PSA, it is expected that a greater fraction of men in the testosterone arm of the study will exceed a PSA threshold, prompting prostate biopsy counseling. As previously noted, prostate cancers are highly prevalent in aging men. About 15% of men whose PSA was initially 3.0 ng/mL or less who undergo 6-core prostate biopsy within a period of 7 years will be found to have prostate cancer; with current 10-12 core biopsies, this rate would likely be higher.

Most prostate cancers detected in men with lower PSA levels as expected in this study will be low-grade and low-volume tumors, tumors that are very common in aging men and are often found at prostate biopsy. It is generally acknowledged that these tumors pose a low risk to aging men; for low-risk (Gleason 3+3) tumors, with active surveillance, 15-year disease specific survivals of 96.7% are achieved (39). For such low-grade tumors, with very high disease-specific survivals achieved without initial treatment, practice guidelines emphasize the importance of active surveillance as an option and the preferred management for many older men (40). Given that side effects and complications (e.g., sexual, urinary, bowel) are an order of magnitude greater with treatment for localized prostate cancer than long-term disease mortality risk, for low-risk cancers, surveillance is often preferred, emphasizing not only the low-risk of these tumors but the minimal impact of their diagnosis on the patient's health.

More recent data are emerging that slightly higher-grade tumors, specifically Gleason 3+4 tumors, have only a modestly-greater risk of disease progression. Among 945 patients with prostate cancer followed on surveillance, 213 were considered of 'intermediate-risk': these included 102 patients with Gleason 3+4 disease and 20 with Gleason 4+3 disease. The other manners by which patients could be classified as intermediate risk were to have PSA levels of 10-15 ng/mL or higher-stage tumors (39). Despite the inclusion of 20 patients with Gleason 4+3 disease, 15-year prostate cancer-specific survival in patients managed with active surveillance was 88.5%. In studies of patients undergoing radical prostatectomy, the proportion of Gleason pattern 4 disease was directly related to the risk of adverse pathology features (associated with risk of recurrence) and directly related to biochemical

recurrence. Emphasizing the difference between a Gleason 3+4 tumor and a 4+3 tumor on biopsy, the risk of adverse pathology and biochemical recurrence, respectively, for biopsies indicating 20-39.9% pattern 4 disease were 5.8% and 43%, respectively. Conversely, for biopsies indicating 60-79.9% pattern 4 disease, the risks were 18% and 81.6%, respectively. The risks of biochemical recurrence for small tumors with low amounts of pattern 4 disease (e.g., 1-9.9%) were 1.5% and 19.6%, respectively (41).

These data, in aggregate, emphasize the potential harms of pursuing prostate biopsy for some men with suspicious PSA levels. Prostate biopsy itself carries a small risk of bleeding but an increasing risk of infection, especially with the emergence of fluoroquinolone-resistant organisms. The risk of infection or sepsis, serious enough for hospitalization, is as high as 3.1% (42). Additionally, given the limited evidence that low-grade prostate cancers pose a risk of morbidity or mortality to the host, even if surveillance for these tumors is pursued with a goal of preventing the complications of treatment, active surveillance is not without risk and harms. Surveillance generally calls for regular (e.g., quarterly or semi-annual) PSA measurements and physical examinations. Additionally, regular (one year following initial diagnosis and every-other year thereafter) prostate biopsies carry the risk of infection and bleeding as well as pain and significant cost.

These data, combined with current prostate cancer management guidelines, suggest the following conclusions for men followed on the TRAVERSE clinical trial:

1. If a man undergoes prostate biopsy and is found to have a high-grade tumor, this is the specific individual who would be most likely to benefit from diagnosis. Additionally, as this tumor’s biologic potential would be the greatest, this outcome would be most important to understand in the two study arms of the TRAVERSE clinical trial. If similar rates of high-grade tumors are noted in the two study arms, a difference in prostate cancer mortality and morbidity would be unlikely.

2. If a man undergoes a prostate biopsy for an elevated PSA and the biopsy is negative for cancer, there is a net harm experienced. The harm includes pain, cost, anxiety, risk of bleeding, infection or hospitalization.

3. If a man undergoes a prostate biopsy for an elevated PSA and the biopsy reveals a cancer that is either low-grade (Gleason 3+3) or lower-risk intermediate-grade (Gleason 3+4), there is likely to be a net harm experienced. While there is a potential that in a small fraction of these men, the cancer diagnosed would have otherwise progressed asymptomatically, causing morbidity and/or mortality and that the diagnosis would allow for an intervention (e.g., surgery or radiation) sufficiently early to allow for a cure, in most men, the diagnosis will not prevent morbidity nor mortality. Additionally, the net negative impact of diagnosis (anxiety, burden of follow up if on surveillance, side effects and cost if treatment is initiated) would argue a net harm of diagnosis.

Given the discussion above, the primary endpoint of the study was selected to be Gleason 4+3 tumors or higher grade.

As the volume of tumor and percent of pattern 4 in tumors diagnosed in the study will be captured through central pathology review, it will be possible to compare rates of prostate cancers including not only high-grade tumors but intermediate-grade tumors of with higher-fractions of Gleason pattern 4 disease (e.g., Gleason 3+4 tumors with > 10% pattern 4, etc).

6. SCHEDULE OF PROSTATE MONITORING DURING THE INTERVENTION PERIOD

- PSA level at baseline, 3 months, 12 months, and then annually
- Digital prostate examination at baseline, 12 months, 3 years and end of the trial; a prostate examination by the participant’s primary care provider or private urologist within the past 6 months would be acceptable provided a source document establishing the date and findings of the examination are available.
- Lower urinary tract symptoms using IPSS at baseline, 3 months, 12 months, 3 years and end of the trial

Assessment	baseline	3 mo	12 mo	24 mo	36 mo	48 mo	60 mo
PSA	X	X	X	X	X	X	X
IPSS	X	X	X		X		X

DRE*	X		X		X		X

*, a DRE performed by the participant's PCP or urologist within six months of the screening date is acceptable provided there is a documented report in the source document. During the intervention period, a DRE performed by the participant's PCP or urologist within 2 months of the scheduled date is acceptable provided there is a documented report in the source document.

If a significant PSA elevation (defined below) is found, the participant will be asked to have a repeat PSA level performed no sooner than 4 weeks after the initial test because a number of PSA elevations are due to assay variability or benign causes that may be associated with transient elevations of PSA. In the T Trial, about 50% of men with confirmed PSA increases that resulted in urological referral had acceptable PSA values when repeated by the urologists about one month later (31). During this period, the participants will be advised to continue their study medication.

7. CRITERIA FOR REFERRAL FOR UROLOGICAL EVALUATION FOR CONSIDERATION OF PROSTATE BIOPSY

A participant will be referred for urological evaluation for consideration of a prostate biopsy if he meets any of the following criteria: (add 5-ari guidelines too)

1. Confirmed Increase >1.4 ng/mL above baseline
2. Detection of a new prostate nodule or induration
3. Absolute PSA value >4.0 ng/mL at any time during the trial

In men receiving steroid 5 alpha reductase inhibitors, a confirmed increase of 0.7 ng/mL or an absolute increase above 2 ng/mL will lead to referral.

Rationale for the criteria for urological referral

A standardized, monitoring plan is necessary to achieve the dual goals of facilitating early detection of adverse prostate events and to prevent unnecessary prostate biopsies that might lead to detection of subclinical prostate cancer. A difficult issue in the follow-up of hypogonadal men receiving testosterone therapy relates to the criteria that should be used to guide the decision to perform prostate biopsy. PSA measurements have considerable test-retest variability (32-34). Transient PSA elevations may be due to other prostatic disorders (32-34). PSA levels may be increased by prostatitis, benign prostatic hyperplasia, prostate trauma, urinary tract infections, prostate cancer, and assay variability (32-34). Nearly 50% of transient PSA elevations will resolve without intervention when the PSA levels are repeated more than 4 weeks after the initial test. Therefore, PSA elevations must be confirmed by repeating the test no sooner than 4 weeks after the initial test.

The 90% confidence limit for the change in PSA levels between two tests performed 3 to 6 months apart in a study of men with benign prostatic hyperplasia was 1.4 ng/ml (35). In a systematic review, the average PSA increase after initiation of testosterone therapy was 0.3 ng/ml in young, hypogonadal men and 0.44 ng/ml in older men (28). The increases in PSA levels after testosterone therapy in androgen-deficient men in excess of 1.4 ng/ml over a 3- to 6-month period are unusual. In the TTrials PSA increases >1.4 ng/mL occurred in 2.4% of participants at 3 months and 4.7% at 12 months in the testosterone group and 1.6% and 0.6% in the placebo group (31). These considerations lead us to suggest urological consultation for evaluation of confirmed PSA increments greater than 1.4 ng/ml after initiation of testosterone therapy. In the T Trial <5% of men 65 and older had a confirmed PSA increase >1.4 ng/ml at one year (31).

8. PROSTATE ENDPOINT ADJUDICATION

The following endpoints will be adjudicated by a Prostate Endpoints Adjudication Committee:

- Prostate cancer status and Gleason score
- Acute urinary retention events
- Invasive prostate surgical procedure (prostatectomy, transurethral prostate resection, or other prostate surgical procedure) for obstruction

Definitions of Prostate Endpoints that Require Adjudication

1. Prostate Cancer

The diagnosis of prostate cancer is based on the evaluation of prostate biopsies and all prostate procedures that yield tissue and which are performed during the duration of the trial, including TURP and prostatectomy. Although great effort will be made to obtain materials to be reviewed by the TRAVERSE Prostate Adjudication Center at the University of Colorado, if the slides cannot be obtained for central pathology review, the local site pathology report will be reviewed by the TRAVERSE Prostate Adjudication Center at the University of Colorado and the diagnosis and Gleason score reported by the local site pathologist will be used as the endpoint.

High grade prostate cancer will be defined as a Gleason score of 4+3 or higher.

2. Acute Urinary Retention

Acute urinary retention (AUR) is the inability to voluntarily pass urine, requiring a visit to the emergency department and/ or placement of a catheter to relieve it, ascertained by participant self-report and verified by review of medical record.

3. Invasive prostate procedure for benign prostatic hyperplasia

An invasive prostate procedure is any surgical procedure on the prostate such as transurethral prostatectomy or open, laser, or incisional prostatectomy for benign prostatic hyperplasia other than a prostate biopsy, ascertained from medical records.

Prostate Cancer Adjudication Committee

The Prostate Cancer Adjudication Committee will include the following:

- Michael O'Leary (Chair)
- Scott Lucia (Designated Prostate Pathologist)
- Kelly Parsons

Adjudication Procedures

The prostate cancer adjudication will be performed by Dr. Scott Lucia at the University of Colorado, Denver, CO. Dr. Lucia will review all biopsies, cytology, and surgical pathology specimens, as well as data from medical records and patient questionnaires to confirm the diagnosis of prostate cancer, using procedures similar to those used previously in the SELECT and the Prostate Cancer Prevention Trial (PCPT). Dr. Lucia and his staff will be blinded to the randomization arm.

All prostate biopsies or cytology specimens performed on the study participants as well as all surgical pathology specimens from any type of prostate surgery performed during the course of the trial will be shipped by an overnight courier to the Central Pathology Laboratory for the TRAVERSE Trial, led by Dr. Scott Lucia at the University of Colorado. The Central Pathology Laboratory staff will have no access to the randomization codes.

Prostate biopsies and prostate surgical procedures may be triggered in one of two ways during the course of the study. First, the study protocol will include pre-specified criteria for the referral of study participants for urologic consultation and consideration of biopsy. Second, the prostate biopsies and surgeries may also be performed independently of the study protocol during the course of participant's usual medical care unrelated to the study; these events will come to the team's attention during the study visits or may be reported by the participants to the study team on their own. These biopsies and surgical specimens will have been processed and read locally as a part of the routine patient care. The study team will request from the participant's health care provider the biopsy, cytology, and/or surgical pathology specimens or representative slides to be read centrally. All biopsies, cytology, and/ or surgical pathology specimens will be reviewed by Dr. Scott Lucia, who will remain blinded to the treatment assignments. After review of the pathology specimens and slides, these materials will be returned to the local site pathologist.

The pathologist will use a diagram in which the biopsies are indexed and localized on the diagram. The following diagnoses will be ascertained for each biopsy core:

- Prostate cancer yes/ no
- Gleason scores (Primary + secondary Gleason grade according to ISUP 2005 and 2014 standards)

- Primary endpoint classification (High grade / low grade)

The pathologist will also estimate the number and percent of each core biopsy containing cancer. The diagnosis and the Gleason scores that Dr. Lucia records will be the one used in the prostate cancer analyses. In the event, the diagnosis (cancer yes/no) or the Gleason score assigned by Dr. Lucia differ from those assigned by the local pathology laboratory, the biopsy material will be read by a second expert genitourinary pathologist at the University of Colorado. The final diagnosis will be by consensus. The general approach for the adjudication of prostate cancer diagnosis is summarized in the table below.

Table: General Approach for Adjudication of Prostate Cancer Diagnosis					
Scenario	Tissue	Local Pathology Report	Central Reading by Scott	Other Source Documents	Adjudication
1	Slides sent Centrally	Cancer and grade assigned locally	Confirms cancer<br>Concurs with Grade	n/a[2]	Central cancer and grade used
2	Slides sent centrally	Local pathology report not available	Cancer present, grade assigned	n/a[1]	Central cancer and grade used
3	Slides sent Centrally	Cancer and grade assigned locally	Either disagrees with cancer presence or change in grade	n/a[1]	Central cancer (yes/no) diagnosis <u>and</u> grade used for the study endpoint
4	Slides sent centrally	No cancer noted	Concurs – no cancer	n/a[1]	No cancer – benign
5	Slides sent centrally	Local pathology report not available	No cancer noted	n/a[1]	No cancer – benign
6	Slides sent centrally	Local pathology calls cancer	No cancer noted	n/a[1]	No cancer - benign
7	Slides not centrally sent	Cancer and grade assigned	Cannot be performed	n/a[1]	Cancer and grade information abstracted[3] and used as endpoint
8	Slides not centrally sent	Local pathology report not available	Cannot be performed	Cancer noted in another source document (e.g., autopsy, clinical note)	Adjudication Committee activated[4]

[2] Other source documents are n/a in the adjudication process <u>unless</u> they are discrepant with the local pathology findings. If so, the adjudication committee would need to review the case. For example, if in scenario 7, the pathology report states "cancer present" but source documents are inconsistent (e.g., a progress note states that cancer was present on a local pathology report but it was sent out for a second opinion and was felt to be negative for cancer), the adjudication committee should become involved.

[3] In this case, an adjudication committee (e.g., clinicians – urologists, medical oncologists, etc) are not necessary. I would recommend that Dr. Lucia or his designee at U Colorado use the pathology report to abstract as much information as possible into the fields for the central pathology report. That way, at least some of the components for analysis can be maintained (e.g., Gleason primary grade, secondary grade, number of positive cores, % of core involved, etc)

[4] This one situation in which the adjudication committee would be necessary. A team of clinicians and at least one pathologist should be involved in examining the source document(s) related to the histologic diagnosis. As much information that could be used to populate fields from the U Colorado pathology should be abstracted. I would recommend that Dr. Lucia be involved in this as he would be the best person to ensure that the data elements are properly selected for his fields.

9	Slides not centrally sent	Local report calls ‘no cancer’	Cannot be performed	*n/a*[1]	No cancer - benign

The Informed Consent will include a statement that the participant agrees to request that slides from all prostate tissue as well as the associated medical records be provided to the Prostate Endpoints Adjudication Committee’s pathologist for adjudication.

The endpoints related to benign prostatic hyperplasia will be similar to those used previously in randomized trials of alpha adrenergic blockers and steroid 5-alpha reductase inhibitors, and will include the change from baseline in International Prostate Symptom Score, the proportions of men who were initiated *de novo* on alpha-blocker or 5-alpha reductase therapy, had acute urinary retention, or underwent any type of prostate surgery related to benign prostatic hyperplasia. The ascertainment of the use of medications, urinary retention and prostate surgical procedure will be based on review of medical records by the Prostate Endpoints Adjudication Committee.

Questions that the study staff at each site will ask the participants during each study visit

Since your last visit:

- Have you had a PSA done outside of this trial? What was the value?
- Have you had a DRE done? What was the result?
- Were you recommended to have a prostate biopsy?
- Did you have one? If not, why not?
- Have you been diagnosed with prostate cancer?[*]
- Have you had any other prostate procedure done?[#]

*If the participant reports that he has undergone a prostate biopsy or been diagnosed with prostate cancer, then additional procedures to retrieve the medical records and biopsy slides need to be activated.

[#]If the participant reports that a prostate procedure was performed since the last visit, the study staff will record the name of the procedure, date of the procedure and the location of the medical facility where the procedure was done. Medical records will be requested to ascertain the procedure and the diagnosis.

[&]If the participant reports that a new prostate medicine was started for any prostate problem, the names and doses of the medicines and the date started should be recorded. The condition for which the medicine was started should also be recorded.

9. STATISTICAL ANALYSES

The pre-specified prostate endpoints will be tabulated by intervention arm. Our primary analyses will be concerned with the likelihood of diagnosis of high grade prostate cancer (Gleason score of 4 + 3 or higher). Secondary endpoints include the diagnosis of lower-grade prostate cancer; prostatic biopsy; acute urinary retention; invasive prostate surgical procedure (prostatectomy, transurethral prostate resection, or other prostate surgical procedure) for benign prostatic hyperplasia; pharmacological therapy for lower urinary tract symptoms (steroid 5 alpha reductase inhibitors or alpha adrenergic blockers); change from baseline in lower urinary tract symptoms, as ascertained using the International Prostate Symptom Score (IPSS); and change in PSA levels from baseline to end of intervention, and comparison of pathologic features in biopsies between arms.

The disparity in probability of diagnosis of high-grade cancer in testosterone vs. placebo will be estimated using a difference of sample proportions and a corresponding 95% confidence interval. The statistical significance of this difference will be assessed using Fisher’s exact test. Analyses of secondary outcomes will utilize a proportional hazards regression model for discrete time. The estimated effect of testosterone and its 95% two-sided confidence interval will be extracted from the model adjusted for age. Statistical tests comparing two treatment arms will be supported by log-rank test and Kaplan-Meier estimates of the incidence function (cumulative event rates over time) obtained for each intervention group. Potential confounding factors will be considered in sensitivity analyses for secondary outcomes however these analyses will be contingent on sufficient number of events that occurred throughout the study duration.

The treatment effect on change in PSA and IPSS score will be estimated using a mixed-effects regression model with repeated measures, where baseline measurement, treatment, visits and treatment-by-visit interaction terms will be treated as fixed effects. This model will be used to generate estimates of differences to one year and to the end of the intervention phase, and will allow for a flexible (i.e. nonlinear) pattern of change in outcomes that may differ according to treatment. The difference between effects of testosterone intervention will be estimated from the model using treatment contrasts and associated 95% confidence intervals.

For pathologic features of biopsies, we will compare number of cores positive for prostate cancer, and linear extent of disease between the two arms among those men for whom tissue samples are submitted. Because the sample size will be modest, this comparison will be primarily descriptive. However, a Wilcoxon rank sum test will be employed to formally compare the groups.

Exploratory analyses for the assessment for potential of ascertainment bias

As noted above, we hypothesize that PSA elevations due to testosterone therapy can lead to prostate biopsy and hence greater apparent incidence of lower-grade cancers that would otherwise have gone undetected. In TRAVERSE, the anticipated combination of similar rates of higher-grade cancers in each arm and equal to greater rates of lower-grade cancers in the testosterone arm would be consistent with this hypothesis. To assess the potential magnitude of the influence of ascertainment on incidence rates, we will determine the adjusted rate of lower and higher grade cancers among men with biopsy, controlling for PSA levels. In this model, the proportion of men in testosterone arm with diagnosis of lower-grade cancer is expected to be equal or lower than the corresponding proportion in placebo after statistical control for PSA levels. Additionally, we will compare for each arm the deviation from age- and race-adjusted predicted incidence of lower and higher grade cancers that would be obtained using the Prostate Cancer Risk Trial risk calculator. We will then evaluate whether the deviation in the observed rates of high grade and low grade cancers versus those predicted by the calculator is greater in the testosterone arm compared to the placebo arm. Owing to the modest absolute number of anticipated incident cancers in TRAVERSE, this analysis will necessarily be exploratory. It will be presented in publication for completeness, with an emphasis on its exploratory nature.

10. STUDY PROCEDURES TO ENSURE INFORMED PATIENT DECISION-MAKING

All organizations with recommendations for PSA screening for prostate cancer recommend informed patient decision-making for screening and for diagnosis of prostate cancer. Perhaps because of the complexity of issues surrounding PSA screening and biopsy, in the busy clinical environment, there is strong evidence that fully-informed patient decision-making is unusual (43). In the arena of decision-to-biopsy for patients with elevated PSA, risk assessment tools that provide individualized risk assessment for biopsy outcomes, permit provision of detailed information to patients that integrate the information in this discussion. Using the extensively-validated Prostate Cancer Prevention Trial Risk Calculator, patients can be provided with estimates of risks of the three biopsy outcomes: no cancer, low-grade (Gleason 3+3) cancer, and higher-grade (Gleason 3+4 and greater) cancer (44). A method to use this tool clinically to enable patients who are at the greatest risk of high-grade disease to opt for biopsy while ensuring that lower-risk patients understand their lower likelihood of benefit for biopsy to understand their decisions, has been advocated (45).

In the TRAVERSE clinical trial, the enrolled middle-aged and older men will be undergoing regular PSA testing, both to help understand the risk of prostate cancer but also for clinical safety reasons. In many of these men, elevations in PSA will be due to the age-related increase of PSA. In many others, if biopsy were performed, tumors would be detected that will ultimately not become clinically-evident in their lifetimes. As such, their participation in the trial may increase their risk of detection of inconsequential prostate cancers with the potential harms of such diagnosis. Additionally, if the hypothesis that increases in PSA in the testosterone arm of the study are unrelated to risk of consequential prostate cancers, there is a risk that an increase in numbers of biopsies in this study arm may lead to an additional net harm to participants (unnecessary biopsies, diagnoses, and treatments).

To both mitigate these risks as well as to ensure that the men who are most likely to harbor high-risk cancers do indeed undergo prostate biopsy, the following process is proposed:

1. For men whose PSA increases by 1.4 ng/mL or more or whose PSA exceeds 4.0 ng/mL, the first step taken will be to repeat the value at least 4 weeks later.

2. For men whose repeat PSA confirms the 1.4 ng/mL increase or a level of 4.0 ng/mL or greater, their risk variables (mention what they are?) will be entered into the PCPT Risk Calculator Version 2.0. The three resulting

estimates will be provided: Risk of no cancer, risk of low-grade cancer, risk of high-grade cancer. These results will be calculated centrally and provided to the patient in numerical and graphical form. (**Exhibit A**). With the patient's own risk estimates, he will then be requested to watch a video (either a DVD provided or an on-line video) that provides extensive and updated information about pros and cons of a prostate biopsy. If, after watching the video, the subject decides he would like a urology referral, this will be arranged by the study site. If the subject demurs, he will continue to undergo PSA testing and, if continued elevations in PSA are noted, he could re-consider possible biopsy at a later date.

11 REFERENCES

1. Liverman CT, Blazer DG. Testosterone and aging, clinical research directions. Washington, DC: National Academies Press, 2004.
2. Bhasin S, Cunningham GR, Hayes FJ, Matsumoto AM, Snyder PJ, Swerdloff RS, Montori VM; Task Force, Endocrine Society. Testosterone therapy in men with androgen deficiency syndromes: an Endocrine Society clinical practice guideline. J Clin Endocrinol Metab. 2010;95(6):2536-59.
3. Wang C, Nieschlag E, Swerdloff R, Behre HM, Hellstrom WJ, Gooren LJ, Kaufman JM, Legros JJ, Lunenfeld B, Morales A, Morley JE, Schulman C, Thompson IM, Weidner W, Wu FC; International Society of Andrology (ISA).; International Society for the Study of Aging Male (ISSAM).; European Association of Urology (EAU).; European Academy of Andrology (EAA).; American Society of Andrology (ASA). Investigation, treatment, and monitoring of late-onset hypogonadism in males: ISA, ISSAM, EAU, EAA, and ASA recommendations. J Androl. 2009;30:1-9.
4. Fowler Jr JE, Whitmore Jr WF. The response of metastatic adenocarcinoma of the prostate to exogenous testosterone. J Urol 1981;126:372–375.
5. Roddam AW, Allen NE, Appleby P, Key TJ. Endogenous sex hormones and prostate cancer: a collaborative analysis of 18 prospective studies. J Natl Cancer Inst. 2008;100(3):170-83
6. Nomura AMY, Heilbrun LK, Stemmermann GN, Judd HL. Prediagnostic serum hormones and the risk of prostate-cancer. Cancer Res. 1988;48:3515-3517.
7. Hsing AW, Comstock GW. Serological precursors of cancer—serum hormones and risk of subsequent prostate-cancer. Cancer Epidemiol Biomarkers Prev. 1993;2:27-32.
8. Carter HB, Pearson JD, Metter EJ, et al. Longitudinal evaluation of serum androgen levels in men with and without prostate-cancer. Prostate. 1995;27:25-31.
9. Gann PH, Hennekens CH, Ma J, Longcope C, Stampfer MJ. Prospective study of sex hormone levels and risk of prostate cancer. J Natl Cancer Inst. 1996;88:1118-1126.
10. Nomura AMY, Stemmermann GN, Chyou PH, Henderson BE, Stanczyk FZ. Serum androgens and prostate cancer. Cancer Epidemiol Biomarkers Prev. 1996;5:621-625.
11. Vatten LJ, Ursin G, Ross RK, et al. Androgens in serum and the risk of prostate cancer: a nested case-control study from the Janus serum bank in Norway. Cancer Epidemiol Biomarkers Prev. 1997;6:967-969.
12. Dorgan JP, Albanes D, Virtamo J, et al. Relationships of serum androgens and estrogens to prostate cancer risk: results from a prospective study in Finland. Cancer Epidemiol Biomarkers Prev. 1998;7:1069-1074.
13. Heikkila R, Aho K, Heliovaara M, et al. Serum testosterone and sex hormone-binding globulin concentrations and the risk of prostate carcinoma—a longitudinal study. Cancer. 1999;86:312-315.
14. Mohr BA, Feldman HA, Kalish LA, Longcope C, McKinlay JB. Are serum hormones associated with the risk of prostate cancer? Prospective results from the Massachusetts Male Aging Study. Urology. 2001;57:930-935.
15. Chen C, Weiss NS, Stanczyk FZ, et al. Endogenous sex hormones and prostate cancer risk: a case-control study nested within the carotene and retinol efficacy trial. Cancer Epidemiol Biomarkers Prev. 2003;12:1410-1416.
16. Stattin P, Lumme S, Tenkanen L, et al. High levels of circulating testosterone are not associated with increased prostate cancer risk: a pooled prospective study. Int J Cancer. 2004;108:418-424.
17. Parsons JK, Carter HB, Platz EA, Wright EJ, Landis P, Metter EJ. Serum testosterone and the risk of prostate cancer: potential implications for testosterone therapy. Cancer Epidemiol Biomarkers Prev. 2005;14:2257-2260.
18. Platz EA, Leitzmann MF, Rifai N, et al. Sex steroid hormones and the androgen receptor gene CAG repeat and subsequent risk of prostate cancer in the prostate-specific antigen era. Cancer Epidemiol Biomarkers Prev. 2005;14:1262-1269.

19. Severi G, Morris HA, MacInnis RJ, et al. Circulating steroid hormones and the risk of prostate cancer. Cancer Epidemiol Biomarkers Prev. 2006;15:86-91.

20. Travis RC, Key TJ, Allen NE, et al. Serum androgens and prostate cancer among 643 cases and 643 controls in the European Prospective Investigation into Cancer and Nutrition. Int J Cancer. 2007;121:1331-1338.

21. Shaneyfelt T, Husein R, Bubley G, Mantzoros CS. Hormonal predictors of prostate cancer: a meta-analysis. J Clin Oncol. 2000;18:847-853.

22. Thompson IM, Pauler DK, Goodman PJ, et al. Prevalence of prostate cancer among men with a prostate-specific antigen level < or =4.0 ng per milliliter. N Engl J Med. 2004;350(22):2239-46.

23. Morgentaler A, Bruning CO 3rd, DeWolf WC. Occult prostate cancer in men with low serum testosterone levels. JAMA. 1996;276(23):1904-6;

24. Schatzl G, Madersbacher S, Thurridl T, Waldmüller J, Kramer G, Haitel A, Marberger M. High-grade prostate cancer is associated with low serum testosterone levels? Prostate. 2001;47(1):52-8;

25. Hoffman MA, DeWolf WC, Morgentaler A. Is low serum free testosterone a marker for high grade prostate cancer. J Urol. 2000;163(3):824-7).

26. Muller RL(1), Gerber L, Moreira DM, Andriole G, Castro-Santamaria R, Freedland SJ. Serum testosterone and dihydrotestosterone and prostate cancer risk in the placebo arm of the Reduction by Dutasteride of Prostate Cancer Events trial. Eur Urol. 2012;62(5):757-64.

27. Pierorazio PM, Ferrucci L, Kettermann A, Longo DL, Metter EJ, Carter HB. Serum testosterone is associated with aggressive prostate cancer in older men: results from the Baltimore Longitudinal Study of Aging. BJU Int. 2005105:824-9.

28. Calof, O.M. et al. Adverse events associated with testosterone replacement in middle-aged and older men: a meta-analysis of randomized, placebo-controlled trials. J Gerontol A Biol Sci Med Sci. 2005;60:1451-7.

29. Fernandez-Balsells, M.M. et al. Clinical review 1: Adverse effects of testosterone therapy in adult men: a systematic review and meta-analysis. J Clin Endocrinol Metab. 2010; 95: 2560-75.

30. Bhasin S, Singh AB, Mac RP, Carter B, Lee MI, Cunningham GR. Managing the risks of prostate disease during testosterone replacement therapy in older men: recommendations for a standardized monitoring plan. J Androl. 2003; 24:299–311.

31. Snyder PJ, Bhasin S, Cunningham GR, Matsumoto AM, et al. Testosterone Trials Investigators. Effects of Testosterone Treatment in Older Men. N Engl J Med. 2016;374:611-24.

32. Riehmann M, Rhodes PR, Cook TD, Grose GS, Bruskewitz RC. Analysis of variation in prostate-specific antigen values. Urology 1993;42:390–397.

33. Scardino PT 2007 The responsible use of antibiotics for an elevated PSA level. Nat Clin Pract Urol. 2007; 4: 1

34. Kobayashi M, Nukui A, Morita T. Serum PSA and percent free PSA value changes after antibiotic treatment. A diagnostic method in prostate cancer suspects with asymptomatic prostatitis. Urol Int. 2008; 80:186–192

35. Gormley GJ, Stoner E, Bruskewitz RC, Imperato-McGinley J, Walsh PC, McConnell JD, Andriole GL, Geller J, Bracken BR, Tenover JS, Vaughan ED, Pappas F, Taylor A, Binkowitz B, Ng J. The effect of finasteride in men with benign prostatic hyperplasia. The Finasteride Study Group. N Engl J Med. 1992; 327:1185–1191.

36. Linthicum, MD: American Urological Association Education and Research, Inc. Prostate-specific antigen best practice statement: 2009 update. 2009.

37. Richie JP, Catalona WJ, Ahmann FR, Hudson MA, Scardino PT, Flanigan RC, deKernion JB, Ratliff TL, Kavoussi LR, Dalkin BL, Waters WB, MacFarlane MT, Southwick PC. Effect of patient age on early

detection of prostate cancer with serum prostate-specific antigen and digital rectal examination. Urology. 1993; 42:365–374.

38. Gosselaar C, Roobol MJ, Roemeling S, de Vries SH, Cruijsen-Koeter I, van der Kwast TH, Schröder FH. Screening for prostate cancer without digital rectal examination and Screening for Prostate Cancer (ERSPC), Rotterdam. Prostate. 2006; 66:625–631

39. Musunuru HB, Yamamoto T, Kotz L, et al. Active surveillance for intermediate risk prostate cancer: Survival outcomes in the Sunnybrook Experience. J Urology 2016 December Epub ahead of print

40. Chen RC, Rumble RB, Loblaw DA, et al. Active surveillance for the management of localized prostate cancer (Cancer Care Ontario Guideline) American Society of Clinical Oncology Clinical Practice Guideline Endorsement. Journal of Clinical Oncology 2016;34:2182-90.

41. Cole AI, Morgan TM, Spratt DE, et al. Prognostic value of percent Gleason Grade 4 at prostate biopsy in predicting prostatectomy pathology and recurrence. J Urology 2016;196:405-11.

42. Lu DD, Raman JD. Strategies for prevention of ultrasound-guided prostate biopsy infections. Infection and Drug Resistance 2016;9:161-69.

43. Holmes-Rovner M, Montgomery JS, Rovner DR, et al. Informed decision-making: Assessment of the quality of physician communication about prostate cancer diagnosis and treatment. Medical Decision Making 2015;35:999-1009.

44. Thompson IM, Ankerst DP, Chic C, et al. Assessing prostate cancer risk: results from the Prostate Cancer Prevention Trial. J Natl Cancer Inst 2006;98:529-34.

45. Thompson IM, Leach RJ, Ankerst DP. Focusing PSA testing on detection of high-risk prostate cancers by incorporating patient preferences into decision making). JAMA 2014;312:995-6.

Exhibit A

Example of a risk assessment provided to the patient.

"You have been noted to have either an increase in your PSA or a PSA above 4.0 ng/mL in the TRAVERSE clinical trial. The PSA test is used to help find prostate cancer early, in time to allow for potentially-curative treatments. In order to determine if prostate cancer is present, a prostate biopsy is necessary. While in some men undergoing prostate biopsy, a cancer destined to cause harm will be found in time to allow a cure, for most men, the biopsy will be negative or will detect a slow-growing tumor that would have never been known about in the man's lifetime.

To help you decide whether you would like a referral to see a physician about a possible biopsy, we are providing you with an individualized assessment of what would likely be found at biopsy. The three possible outcomes are <u>no cancer</u>, <u>low-risk cancer</u>, or <u>high-risk cancer</u>. Your individual risk is displayed below. Out of 100 men like you who would undergo biopsy, we would expect 76% to have no evidence of cancer on biopsy. An additional 20% would have a low-risk cancer. About 4% (4 in 100 men) of men would be found to have high-risk cancers that would be more likely to benefit from treatment.

To help you understand whether you would like a referral to see a urologist for a possible biopsy, we ask that you watch the enclosed DVD. Alternatively, you may watch the video on line at: http\\www.demo.com. After you watch the video and consider your own risk, if you desire a prostate biopsy, your TRAVERSE study site will help make you an appointment with a urologist. If you do not desire a biopsy, your PSA will be repeated regularly during the study and your risk can be reassessed at that time."

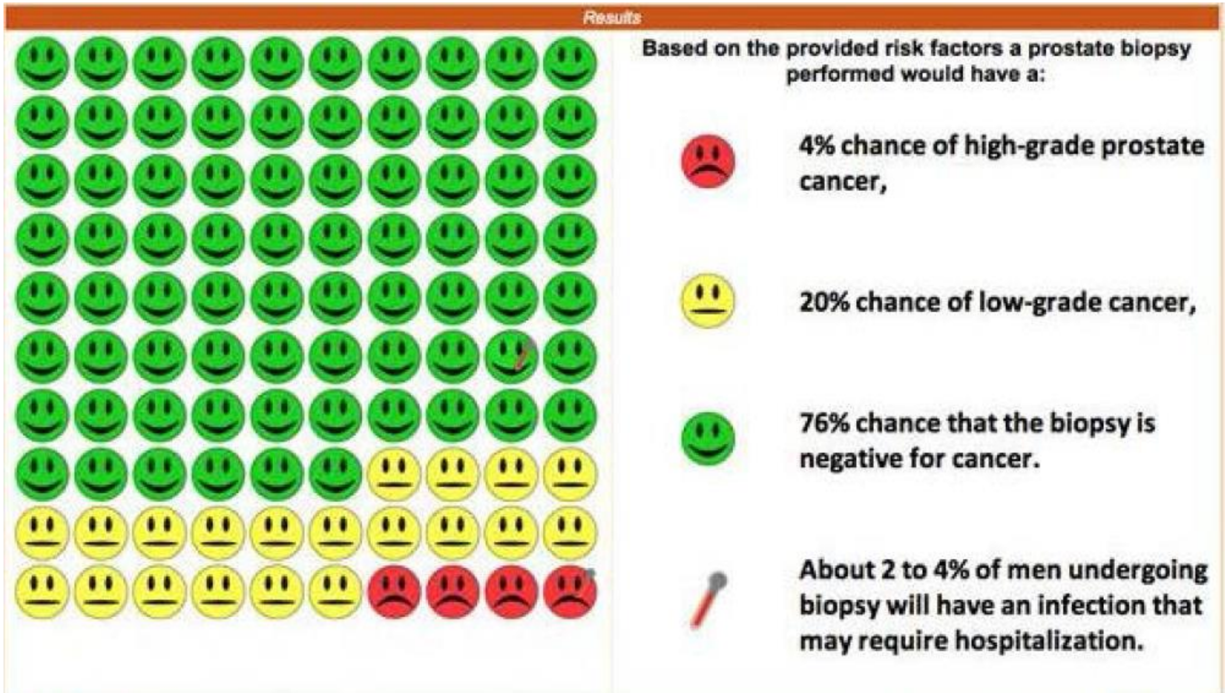

Supplement: Supplement 1. — TRAVERSE Trial Prostate Safety Monitoring Plan [file jamanetwopen-e2348692-s001.pdf]
