# Supplementary material for: Prostate Safety Events During Testosterone Replacement Therapy in Men With Hypogonadism: A Randomized Clinical Trial
Source: JAMA Netw Open. 2023 Dec 27;6(12):e2348692. doi: 10.1001/jamanetworkopen.2023.48692 (PMC10753401; doi:10.1001/jamanetworkopen.2023.48692)
Supplement: Supplement 2. — Statistical Analysis Plan [file jamanetwopen-e2348692-s002.pdf]

Statistical Analysis Plan

Supplement for Analyses of Prostate Safety Endpoints

Study M16-100
Testosterone Replacement Therapy for Assessment of Long-Term Vascular Events and Efficacy ResponSE in Hypogonadal Men (TRAVERSE) Study

Date: January 09, 2022

Table of Contents

1. Introduction 4
2. Study Background 4
3. Objective 4
4. Study Design 5
4.1. Background 5
4.2. Variables Used for Stratification at Randomization 6
5. Endpoints 6
5.1. Primary Endpoint 6
5.2. Secondary Endpoints 6
6. Interim Analysis 7
7. Multiplicity Testing Procedures for Type-I Error Control 7
8. Missing Data Imputation 7
9. Analysis Populations and Important Subgroups 7
9.1. Analysis population 7
9.2. Analysis subpopulations 7
10. Pre-specified Subgroup Analyses 8
11. Analysis Conventions 8
11.1. Definition of Baseline 8
11.2. Definition of Visit Windows 8
11.3. Referral to Prostate Biopsy Procedure 8
11.4. Adjudication of Prostate Endpoints 9
12. Baseline Characteristics, Medical History and Study Drug Exposure 10
12.1. Baseline Characteristics 10
12.2. Report of Treatment Exposure and Compliance 10
13. Analysis of Endpoints 10
13.1. Assessment for Potential of Ascertainment Bias 10
13.2. Primary Analysis 12
13.3. Analysis of Secondary Endpoints 12
14. Changes in this version 14
15. References 15
16. Schedule of Activities 17
17. Efficacy Analysis Time Windows 18

17.1 For Activity: I-PSS 18
17.2 For Activity: PSA 18
17.3 Time Windows for Time to Event Data 19

List of Abbreviations

5-ARI	5-Alpha Reductase Inhibitor
AUR	Acute Urinary Retention
CV	Cardiovascular
CVD	Cardiovascular Disease
DVD	Digital Versatile Disc
ESC	Executive Steering Committee
FAS	Full Analysis Set
I-PSS	International Prostate Symptom Score
IRB	Institutional Review Board
IRT	Interactive Response Technology
MACE	Major Adverse Cardiac Event
MICE	Multiple Imputation by Chained Equations
PCPT	Prostate Cancer Prevention Trial
PDQ	Psychosexual Daily Questionnaire
PSA	Prostate Specific Antigen
SAP	Statistical Analysis Plan
TRT	Testosterone Replacement Therapy
TURP	Transurethral Resection of the Prostate

1. Introduction

This statistical analysis plan (SAP) provides details to elaborate statistical methods for the analyses of prostate safety endpoints collected as outlined in the protocol of Study M16-100 parent trial (Amendment 1 dated 26 February 2018[1]) and to describe analysis conventions to guide the statistical programming work.

All analyses will be performed using SAS Version 9.2 or higher (SAS Institute, Inc., Cary, NC 27513) or R Version 3.4 or higher. This SAP will not be updated in case of future (administrative or minor) amendments to the TRAVERSE trial protocol unless the changes have any impact on the analysis of study data described here.

Analytic approaches described below are presented under the expectation that there will be sufficient information to render them meaningful. However, it is possible that the number of prostate safety events (e.g. new cancers and/or biopsies) may not be large enough to provide sufficient statistical power for some suggested analyses. In this case, only descriptive analyses may be performed.

2. Study Background

3. Objective

Primary Objective:

- To compare the incidence of high-grade prostate cancer (Gleason score of 4 +3 or higher) in middle-aged and older hypogonadal men randomized to TRT or placebo gel.

Secondary Objectives:

- To compare the frequency of prostate biopsies in men treated with TRT and placebo gel.
- To compare the incidence of any prostate cancer in hypogonadal men treated with TRT and placebo gel.
- To compare changes in PSA levels over time in men treated with TRT and placebo.
- To compare the incidence of acute urinary retention in men treated with TRT and placebo gel.
- To compare the percentage of men starting pharmacologic treatment for lower urinary tract symptoms in the TRT and placebo groups.
- To compare the percent of men undergoing invasive prostate surgical procedures (e.g., prostatectomy, transurethral prostate resection, or other prostate surgical procedure) for benign prostatic hyperplasia (BPH) in the TRT and placebo groups.
- To compare the effects of TRT and placebo on change in I-PSS score over the entire study period.

- To compare the percent of men undergoing treatment for prostate cancer (radical prostatectomy, radiation therapy, focal ablative therapy) in the TRT and placebo groups.
- Analyses to assess bias in ascertainment of prostate events

4. Study Design

The TRAVERSE parent trial is a Phase 4, randomized, double-blind, placebo-controlled, multicenter study of topical TRT in symptomatic hypogonadal men with increased risk for CV disease. The initial planned study enrollment is approximately 6,000 subjects based on the projected timing when 256 MACE will occur under initial assumptions of the annual event rate, subject accrual rate, and study discontinuation rate. There will be approximately 400 sites in North America. An Interactive Response Technology (IRT) system will randomize subjects to receive either topical testosterone or placebo in a 1:1 ratio. Randomization will be stratified by pre-existing CV disease (Yes/No). Titration of testosterone dose will occur in subjects receiving active testosterone, while sham dosage titrations will occur in subjects receiving placebo gel via the central IRT system. The Screening Period is up to 60 days prior to first dose of study drug. Once subjects meet all of the eligibility criteria during Screening, they will be randomized (1:1 ratio) to active study drug or placebo and will be followed until the study ends, with stratification by prior CVD event status (see below). Importantly, randomized subjects who elect to discontinue study drug will also be followed until the study ends unless the subject dies or withdraws from the study completely (withdrawal of informed consent) earlier. Subjects who discontinue study drug will still be asked to follow their regularly scheduled protocol visits. Subjects who interrupt study drug will be allowed to restart study drug at any time.

4.1. Background

Evidence of a relationship between TRT and the incidence of prostate cancer is mixed. In the Baltimore Longitudinal Study of Aging, aggressive prostate cancers were reported to be associated with higher levels of total and free testosterone.[1] Also, testosterone administration increases prostate specific antigen (PSA) in hypogonadal men and can promote the growth of metastatic prostate cancer.[3] On the other hand, most population-based studies have not associated high total or free testosterone levels with increased cancer risk,[4-6] and an analysis of the placebo arm from the Prostate Cancer Prevention Trial (PCPT) found no significant associations of total or free testosterone and risk of total, low (Gleason < 7) or high-grade (Gleason 7 – 10) prostate cancer.[7,8] Occult prostate cancer is common in middle-aged and older men, and the prevalence increases with increases in PSA and age.[7,9] The designers of the current study recognize that testosterone therapy usually increases PSA in circulation and thus, in the current study it is likely that more men randomized to testosterone will be referred for prostate evaluation and possible biopsy based on this laboratory finding. Consequently, testosterone-treated men may have an increased risk of detection of subclinical prostate cancer that was present prior to treatment.[5,10,11] This inherent surveillance bias could result in a greater number of prostate biopsies that are positive for low-grade indolent prostate cancers in men randomized

to the testosterone arm than in those randomized to the placebo arm. With this recognition of the inherent detection bias from testosterone treatment leading to the detection of low grade prostate cancers, this study will assess the effect of testosterone and placebo on the development of high grade prostate cancer, defined as Gleason 4 + 3 or greater, as these higher grades are associated with increased morbidity and mortality. **All analyses will consider the potential for bias in ascertainment of events as part of sensitivity assessments.**

4.2. Variables Used for Stratification at Randomization

TRAVERSE trial randomization will be stratified by pre-existing CV disease (Yes/No). It is expected that in the parent trial 30% of the randomized subjects will satisfy inclusion criteria for pre-existing CV disease criteria (secondary prevention), and the remaining 70% will satisfy CV risk factors criteria (primary prevention) combined.

The ESC and Sponsor may decide to cap the primary prevention cohort if that cohort is found to consistently exceed 70% of the total population enrolled or if the pooled primary event rate falls below projections.

5. Endpoints

5.1. Primary Endpoint

- Time to first diagnosis of high-grade prostate cancer (Gleason score of 4 +3 or higher) defined as time from randomization to an event

5.2. Secondary Endpoints

- Incidence of high grade prostate cancer (Gleason score of 4 +3 or higher)
- Incidence of any prostate cancer
- Time to prostate biopsies defined as time from randomization to an occurrence of prostate biopsy
- Comparison of cumulative incidence of one or more biopsies by 3, 12, and 24 months post-randomization
- Time to occurrence of first prostate cancer of any grade
- Time to occurrence of first acute urinary retention defined as time from randomization to an event
- Time to the first occurrence of starting pharmacologic treatment for lower urinary tract symptoms
- Time to the first occurrence of invasive prostate surgical procedures (e.g., prostatectomy, transurethral prostate resection, or other prostate surgical procedure) for benign prostatic hyperplasia
- Change from baseline in PSA levels and I-PSS score with time

- Gleason score and TNM stage of diagnosed prostate cancers
- Proportions of men with prostate biopsy, acute urinary retention, starting new pharmacologic therapy for lower urinary tract symptoms, and invasive prostate surgical procedures (e.g., prostatectomy, transurethral prostate resection,) for benign prostatic hyperplasia will be compared between the two intervention arms.
- Proportions of men undergoing treatment (radiation therapy, radical prostatectomy, focal ablation procedures) for prostate cancer will be compared between the two intervention arms.

6. Interim Analysis

No formal interim analysis or stopping rules are specified. Safety comparisons will be reported to the trial DSMB as stipulated in the parent TRAVERSE trial protocol.

7. Multiplicity Testing Procedures for Type-I Error Control

Type I error adjustments for multiple comparisons are not planned for this sub-study.

There is only one primary treatment comparison for the primary objective.

8. Missing Data Imputation

Following the TRAVERSE protocol, no imputation is planned. Multiple imputation of endpoints and covariates by the MICE methodology[12,13] may be considered where appropriate (i.e. for data reported in the manuscripts). This method is notable for being able to handle clustering of repeated measures at the participant level, a feature of the design of this trial.

9. Analysis Populations and Important Subgroups

9.1. Analysis population

Most analyses will utilize the Full Analysis Set (**FAS**) comprising all subjects eligible for analysis in the main TRAVERSE trial. Subjects will be categorized according to treatment assigned at randomization.

A modified analysis set removing participants from the point at which they are deemed treatment noncompliant by the parent trial may also be considered and used in sensitivity analyses.

9.2. Analysis subpopulations

We will consider those individuals with biopsy in analyses of the rate of diagnoses per biopsy.

10. Pre-specified Subgroup Analyses

As noted above, analyses will be performed only where there are sufficient cases to support them. Under this stipulation, the following subgroup analyses will be considered for the prostate sub study endpoints:

- by race and ethnicity
- by age (e.g. < 65 year, ≥ 65 years)
- by family history of PCa (first degree relatives)
- by baseline PSA (dichotomized at median or other relevant value)
- by baseline total testosterone levels (e.g. < 250 mg/dl, ≥ 250 mg/dl)
- by baseline BPH (those with prevalent diagnoses vs those without)

Additional sub-group analyses may be also carried out as appropriate.

11. Analysis Conventions

11.1. Definition of Baseline

Baseline on each outcome measure will be defined as the last available measurement obtained prior to the first dose of study drug (defined as on or before Day 1) (Protocol Appendix C).

11.2. Definition of Visit Windows

Definitions of the visit windows (baseline and on-treatment) are presented in Section 16 (Tables 16.1-16.3) and schedule of sub-study activities is presented in Section 15.

11.3. Referral to Prostate Biopsy Procedure

A subject will be referred for urological evaluation for consideration of further work-up which may include a prostate biopsy if he meets any of the following criteria:

- Confirmed increase > 1.4 ng/mL above Baseline during the first year [> 0.7 ng/mL in men on 5-Alpha Reductase Inhibitor (5-ARI)]
- Detection of a new prostate nodule or induration
- Confirmed absolute PSA value > 4.0 ng/mL at any time during the study (> 2.0 ng/mL in men on 5-ARI)
- Men 45 – 54 years of age whose Baseline PSA was < 1.5 ng/mL and whose PSA increases to > 3.0 ng/mL at any time during the study

For men aged 55 or older whose repeat PSA confirms the 1.4 ng/mL increase or a level > 4.0 ng/mL, their risk variables will be entered into the PCPT Risk Calculator Version 2.0.[14] The three

resulting estimates will be provided: Risk of no cancer, risk of low-grade cancer, risk of high-grade cancer. These results will be calculated centrally and provided to the site and subject. With the subject's own risk estimates, he may then be provided with an IRB approved video (either a digital versatile disc (DVD) provided or an on-line video) that provides extensive and updated information about pros and cons of a prostate biopsy. Along with the video, the subject will be provided with a urology referral.

The IRT system will communicate the need for the site to refer subjects with confirmed increases in PSA to a urologist for further evaluation. Unblinded PSA results will be provided to the site once a repeat PSA is confirmed to meet the thresholds. It is recommended that study drug be continued in these subjects while being evaluated by a urologist. Subjects with non-confirmatory findings (e.g., negative biopsy, decision on the urologist's part not to do additional investigations) following the evaluation may continue on study drug. Subjects with biopsies positive for prostate cancer should have study drug discontinued but will continue all other trial procedures.

11.4. Adjudication of Prostate Endpoints

The following endpoints will be adjudicated by a Prostate Endpoints Adjudication Committee:

1. Prostate cancer status and Gleason score
2. Acute urinary retention events
3. Invasive prostate surgical procedure (prostatectomy, transurethral prostate resection, or other prostate surgical procedure) for obstruction

The diagnosis of prostate cancer is based on the evaluation of prostate biopsies and all prostate procedures that yield tissue and which are performed during the duration of the trial, including TURP and prostatectomy. Although great effort will be made to obtain materials to be reviewed by the TRAVERSE Prostate Adjudication Center at the University of Colorado, if the slides cannot be obtained for central pathology review, the local site pathology report will be reviewed by the TRAVERSE Prostate Adjudication Center at the University of Colorado and the diagnosis and Gleason score reported by the local site pathologist will be used as the endpoint. High grade prostate cancer will be defined as a Gleason score of 4+3 or higher.

Acute urinary retention (AUR) is the inability to voluntarily pass urine, requiring a visit to the emergency department and/ or placement of a catheter to relieve it, ascertained by participant self-report and verified by review of medical record.

An invasive prostate procedure is any surgical procedure on the prostate such as transurethral prostatectomy or open, laser, insertion of prostatic urethral lift (PUL) or incisional prostatectomy for benign prostatic hyperplasia other than a prostate biopsy, ascertained from medical records.

12. Baseline Characteristics, Medical History and Study Drug Exposure

12.1. Baseline Characteristics

In presenting the FAS we will obtain data from the parent TRAVERSE trial. For presentation of subcohorts (e.g. in subgroup analyses) additional tabular displays may be conducted. Data collected in this sub-study will be documented using summary tables. Statistics for continuous variables will include mean, median, standard deviation, minimum, maximum, and sample size for each treatment group, and two-sided 95% confidence intervals of the mean difference between the treatment groups. Binary variables will be described with frequencies, percentages, and two-sided 95% confidence intervals of the difference in percentages between treatments.

Medical history will mirror the presentation in the parent TRAVERSE trial, and will include at minimum history of depression, CV history; nicotine and alcohol use; and testosterone use.

12.2. Report of Treatment Exposure and Compliance

Compliance statistics will be obtained from the parent trial. Where additional displays are necessary (e.g. in exploratory subgroup analyses), we may compute additional summaries relevant to this sub-study. Continuous summaries of subjects' total duration of treatment with study drug may be generated for multiple time intervals (i.e. cumulative to 1- and 2-year follow-up).

13. Analysis of Endpoints

All analyses will classify individuals according to their randomized assignment.

It is acknowledged here that the total number of events observed may not support all analyses (e.g. regression models) described below. In the case that models are not practicable, descriptive alternatives will be employed.

13.1. Assessment for Potential of Ascertainment Bias

A major goal of this substudy will be to capture the incidence rates and rate ratios quantifying the absolute and relative risks of new prostate cancers in the testosterone and placebo arms of the TRAVERSE trial. Owing to the manner by which cancers will be diagnosed - via biopsy, which in turn may be suggested by post-randomization increase in prostate-specific antigen (PSA) - there is the potential for disproportionate capture of indolent cancers in the testosterone arm. Naïve estimation of rates may therefore provide spurious evidence of increased risk in testosterone arm. Some potential sources of bias, and potential strategies to deal with them, are briefly discussed here

1. Increase in PSA in testosterone arm, relative to placebo, owing strictly to administration of testosterone. We anticipate greater and more rapid increase in PSA levels in T arm observable at 3 and 12 months visits than in the placebo arm. We anticipate this increase will be apparent at 3 months and the increase will have leveled off by 12 months, and the rate of PSA increase thereafter will be similar in the two arms, provided cancer risk is similar in the two arms. We hypothesize that continued differentiation in the rate of increase in PSA beyond one year may be evidence of a higher risk of prostate cancer incidence.

Approach: At each PSA assessment time point, we will estimate median change from baseline and related descriptive statistics by arm. A linear model will be used to estimate the slope of longitudinal PSA for each treatment group allowing for individual random effects and differing slopes before and after Year 1. We will also graphically display the data over time. We will test whether the slope of PSA varies between the first 12 months and the subsequent intervention period, and if the pattern is different by arm.

2. The T group will have more biopsies than the Placebo group within the first year

We expect that exposure to T will cause more men on that arm to cross the PSA threshold (4.0 ng/ml or velocity criterion) and therefore to be recommended for biopsy. The cause for the increase in PSA is likely to be due to one of two causes:

a. An androgen-driven increase in PSA, or
b. Stimulation of pre-existing prostate cancers, i.e., exposure to T via its impact on PSA will identify men who had undetected prostate cancer at study entry.

Approach: We will use the PCPT risk calculator to quantify the baseline risk of prostate cancer and high grade disease for all men entering the trial based on PSA, DRE status, age, race, family history, and prior prostate biopsy status. We will compare patterns of baseline risk and recommendation for biopsy across the two arms, and then compare the actual incidence of cancer among individuals with biopsies relative to their predicted probabilities. Although post-randomization PSA is expected to be slightly higher in the T arm, the relative pattern of risk at baseline and subsequent biopsy recommendation will be the same for both arms conditional on PSA level.

3. There may be more prostate cancers diagnosed in the T arm during the first year compared to Placebo arm, but we anticipate that the incidence will be similar thereafter.

Owing to the greater rate of biopsy resulting from elevated PSA levels, we expect a few more prostate cancers in that arm during the first 12 months compared to placebo. However, we expect the rate of prostate cancer (where the number of men who have a biopsy is the denominator) will be comparable between the two arms after year 1.

Approach: We will descriptively report on men whose PSA and DRE screening data would suggest that they may be candidates for prostate biopsy, with emphasis on age, race and ethnicity, family history of PCa, PSA at baseline, and the PCPT risk calculator. We will report the number of men who undergo biopsy and the result of that test including grade and stage of disease if positive at 3 months, 1 year and subsequent years by treatment arm. We will plot the cumulative incidence of prostate cancer and high-grade disease by treatment arm over the duration of follow-up.

We will likely see a greater number of biopsies in the testosterone group during the first 12 months, but the biopsy rates will be similar thereafter. If testosterone does not induce new prostate cancers, the curve should look quite a bit like the high-grade (artifactually-caused) cancer curve seen in PCPT.

13.2. Primary Analysis

Analyses of high grade prostate cancer occurrence will utilize a proportional hazards regression model for discrete time. The estimated effect of testosterone and its 95% two-sided confidence interval will be extracted from the model adjusted for prior CVD event. Statistical tests comparing two treatment arms will be supported by log-rank test and Kaplan-Meier estimates of the incidence function (cumulative event rates over time) obtained for each intervention group. Potential confounding factors will be considered in sensitivity analyses for secondary outcomes; however, these analyses will be contingent on sufficient number of events occurring throughout the study duration. Models will consider the potential for biases, as noted above, and provide this context in data presentation.

As adjunct to these assessments, we will also consider cumulative incidence of biopsies and cancers at 1 year and during the subsequent intervention period, with estimates obtained from the same regression models described above. We will adopt a parallel approach to consider, among those with biopsy in each arm, the rate of total and high-grade cancers.

13.3. Analysis of Secondary Endpoints

Secondary endpoint of prostate cancer occurrence (any grade) will be analyzed by proportional hazards regression model for discrete time. The estimated effect of testosterone vs. placebo and its 95% two-sided confidence interval will be derived from the model. Statistical tests comparing two treatment arms will be supported by log-rank test and Kaplan-Meier estimates of the incidence function (cumulative event rates over time) obtained for each intervention group. Competing risk of death will be considered as appropriate.

Analyses of other time to event outcomes - prostate biopsy, acute urinary retention, start of pharmacologic treatment for lower urinary tract symptoms and invasive prostate surgical

procedures for benign prostatic hyperplasia - will be conducted in similar fashion to prostate cancer analyses.

Linear mixed model will be employed to compare effect of testosterone intervention on the change over time in PSA levels and IPSS score. Change will be defined as the difference from the baseline value. Models will be adjusted for covariates determined either by inspection to be substantially imbalanced across treatment arms to a degree necessitating adjustment, as determined by combination of numerical imbalance and clinical import of the covariate measure. Models will have as a base set of covariates study visit, treatment effect, visit-by-treatment interaction and baseline value as fixed effects. Random intercept will be included at participants' level. Unstructured covariance matrix will be assumed, however if convergence of the model is not achieved, then a compound symmetry structure will be utilized. Effects of overall change in outcomes over entire treatment period will be calculated as an average score from all visits, and will be extracted, along with two-sided 95% confidence intervals, from the mixed-model framework.

Sensitivity analyses of treatment effect may also be performed where appropriate (i.e. inclusion in the model other stratification factors etc.).

14. Changes in this version

Clarified that descriptive analyses may substitute for modeling approaches if number of observed endpoints are considered insufficient to support models.

15. References

1. Study protocol of M16-100, Amendment 1. 26 February 2018.
2. Pierorazio PM, Ferrucci L, Kettermann A, et al. Serum testosterone is associated with aggressive prostate cancer in older men: results from the Baltimore Longitudinal Study of Aging. BJU Int. 2010;105(6):824-9.
3. Fowler JE Jr, Whitmore WF Jr. The response of metastatic adenocarcinoma of the prostate to exogenous testosterone. J Urol. 1981;126(3):372-5.
4. Mohr BA, Feldman HA, Kalish LA, et al. Are serum hormones associated with the risk of prostate cancer? Prospective results from the Massachusetts Male Aging Study. Urology. 2001;57(5):930-5.
5. Bhasin S, Singh AB, Mac RP, et al. Managing the risks of prostate disease during testosterone replacement therapy in older men: recommendations for a standardized monitoring plan. J Androl. 2003;24(3):299-311.
6. Roddam AW, Allen NE, Appleby P, et al. Endogenous sex hormones and prostate cancer: a collaborative analysis of 18 prospective studies. J Natl Cancer Inst.2008;100(3):170-83.
7. Thompson IM, Pauler DK, Goodman PJ, et al. Prevalence of prostate cancer among men with a prostate-specific antigen level < or =4.0 ng per milliliter. N Engl J Med. 2004;350(22):2239-46.
8. Schenk JM, Till C, Hsing AW, et al. Serum androgens and prostate cancer risk: results from the placebo arm of the Prostate Cancer Prevention Trial. Cancer Causes Control 2016;27(2):175-182.
9. Morgentaler A, Rhoden EL. Prevalence of prostate cancer among hypogonadal men with prostate-specific antigen levels of 4.0 ng/mL or less. Urology.2006;68(6):1263-7.
10. Calof OM, Singh AB, Lee ML, et al. Adverse events associated with testosterone replacement in middle-aged and older men: a meta-analysis of randomized, placebo-controlled trials. J Gerontol A Biol Sci Med Sci. 2005;60(11):1451-7.
11. Fernández-Balsells MM, Murad MH, Lane M, et al. Clinical review 1: Adverse effects of testosterone therapy in adult men: a systematic review and metaanalysis. J Clin Endocrinol Metab. 2010;95(6): 2560-75.
12. Van Buuren, S., Flexible Imputation of Missing Data. 2012, Chapman & Hall/CRC.

13. Van Buuren, S. and K. Groothuis-Oudshoorn, mice: Multivariate Imputation by Chained Equations in R. Journal of Statistical Software, 2011. 45(3): p. 67.
14. Ankerst DP, Hoefler J, Bock S, et al. The prostate cancer prevention trial risk calculator 2.0 for the prediction of low-versus high-grade prostate cancer. Urology. 2014;83(6):1362-7.
15. SAS Institute Inc. SAS/STAT® 13.1 User's Guide. Cary, NC: SAS Institute Inc.; 2013.
16. Zou G. A Modified Poisson Regression Approach to Prospective Studies with Binary Data. American J Epidemiol 2004; 159(7): 702–706.
17. Rivers, C., M.S. Majumder and E.T. Lofgren. Risks of Death and Severe Disease in Patients with Middle East Respiratory Syndrome Coronavirus, 2012–2015. Am J Epidemiol. 2016;184(6): 460-4.

16. Schedule of Activities

Activity	SV1	SV2	SV3	D1 (Baseline)	W 2	M 1 W 4	M 3 W 12	M 6 W 26	M 9 W 39 (phone)	M 12 W 52 (year 1)	M 15 W 65(phone)	M 18 W 78	M 21 W 90 (phone)	M 24 W 104 (year 2)	M 27 W 116 (phone)	M 30 W 130	M 33 W 142 (phone)	M 36 W 156 (year 3)	M 39 W 168 (phone)	M 42 W 182	M 45 W 194 (phone)	M 48 W 208 (year 4)	M 51 W 220 (phone)	M 54 W 234	M 57 W 246 (phone)	M 60 W 260/FV (Year 5)	PD	Unscheduled	30-Day Call
AE Recording	X	X	X	X	X	X	X	X	X	X	X	X	X	X	X	X	X	X	X	X	X	X	X	X	X	X	X	X	X
Prior/Concomitant Medication	X	X		X	X	X	X	X	X	X	X	X	X	X	X	X	X	X	X	X	X	X	X	X	X	X	X	X	X
Site Endpoint Questionnaire Form					X	X	X	X	X	X	X	X	X	X	X	X	X	X	X	X	X	X	X	X	X	X	X	X	X
I-PSS		X		X			X			X								X								X	X		
DRE		X								X								X								X	X	X	
PSA	X						X			X				X				X				X				X	X		
Hematology		X		X				X		X		X		X				X				X				X	X	X	

17. Efficacy Analysis Time Windows

17.1 For Activity: I-PSS

Scheduled Visit	Nominal Day (Study Day)	Time Window (Study Days Range)
Day 1	1	≤ 1
M3	84	2- 224
M12	364	225 - 728
M36	1092	729 - 1456
M60	1820	1457-2180
Final Visit / PD		2 to ≤ 2 days after the last dose of study drug

17.2 For Activity: PSA

Scheduled Visit	Nominal Day (Study Day)	Time Window (Study Days Range)
Day 1	1	≤ 1
M3	84	2 - 224
M12	364	225 - 546
M24	728	547 - 910
M36	1092	911 - 1274
M48	1456	1275 - 1638
M60	1820	1639 - 2180
Final Visit / PD		2 to ≤ 2 days after the last dose of study drug

17.3 Time Windows for Time to Event Data

Scheduled Visit	Nominal Day (Study Day)	Time Window (Study Days Range)
Day 1/Baseline[a]	1	≤ 1
Week 2	14	2 to 21
Week 4	28	22 to 56
Week 12	84	57 to 133
Week 26	182	134 to 227
Week 39	273	228 to 318
Week 52	364	319 to 409
Week 65	455	410 to 500
Week 78	546	501 to 588
Week 90	630	589 to 679
Week 104	728	680 to 770
Week 116	812	771 to 861
Week 130	910	862 to 952
Week 142	994	953 to 1043
Week 156	1092	1044 to 1134
Week 168	1176	1135 to 1225
Week 182	1274	1226 to 1316
Week 194	1358	1317 to 1407
Week 208	1456	1408 to 1498
Week 220	1540	1499 to 1589
Week 234	1638	1590 to 1680
Week 246	1722	1681 to 1771
Week 260 (Final Visit)	1820	2 to ≤ 2 days after last dose of study drug

a. Day of first dose of double-blind study drug.

b. The last value within the window will be used to define Final.

Document Approval

Study M16100 - Statistical Analysis Plan for Prostate Safety Endpoints

Version: 2.3 **Date:** 09-Jan-2022 **Company ID:** 04122018-00F9F683CEE0E1-00001-en

Signed by:	Date:	Meaning of Signature:

08 February 2022

Shalender Bhasin, MD — Date
Co-Principal Investigator
Brigham and Women's Hospital/Harvard Medical School

09 Feb, 2022

Panagiotis Flevaris, MD, Ph.D. — Date
Medical Director
AbbVie Inc.

08 February 2022

Thomas G. Travison, Ph.D. — Date
Biostatistician
Harvard Medical School

08 February 2022

Karol M. Pencina, Ph.D. — Date
Biostatistician
Brigham and Women's Hospital/Harvard Medical School

09 February 2022

Xue Li, PhD — Date
Biostatistician
AbbVie Inc.
