# Supplementary material for: Prostate Safety Events During Testosterone Replacement Therapy in Men With Hypogonadism: A Randomized Clinical Trial
Source: JAMA Netw Open. 2023 Dec 27;6(12):e2348692. doi: 10.1001/jamanetworkopen.2023.48692 (PMC10753401; doi:10.1001/jamanetworkopen.2023.48692)
Supplement: Supplement 3. — eAppendix 1. TRAVERSE Trial Sites and Investigators eAppendix 2. The TRAVERSE Prostate Study Committee eFigure 1. Sensitivity Analysis of the Primary and Major Secondary Prostate Safety Endpoints Using a Cox Proportional Hazards Model eFigure 2. Sensitivity Analysis of the Primary and Major Secondary Prostate Safety Endpoints Using the Same Discrete Proportional Hazards Model as the Primary Analysis but Censoring Events Occurring After One Year Following End of Treatment eFigure 3. Sensitivity Analysis of the Primary and Major Secondary Prostate Safety Endpoints Using the Same Discrete Proportional Hazards Model as the Primary Analysis but Censoring Events Occurring After 30 Days Following End of Treatment eFigure 4. Changes in PSA Levels From Baseline in the TRT and Placebo Groups eTable 1. Maximum Attained PSA Values in ng/mL Prior to Prostate Biopsy for Men Diagnosed With Prostate Cancer eTable 2. Serum Dihydrotestosterone Concentrations [file jamanetwopen-e2348692-s003.pdf]

Supplementary Online Content

Bhasin S, Travison TG, Pencina KM, et al. Prostate safety events during testosterone replacement therapy in men with hypogonadism: a randomized clinical trial. *JAMA Netw Open*. 2023;6(12):e2348692. doi:10.1001/jamanetworkopen.2023.48692

eAppendix 1. TRAVERSE Trial Sites and Investigators
eAppendix 2. The TRAVERSE Prostate Study Committee
eFigure 1. Sensitivity Analysis of the Primary and Major Secondary Prostate Safety Endpoints Using a Cox Proportional Hazards Model
eFigure 2. Sensitivity Analysis of the Primary and Major Secondary Prostate Safety Endpoints Using the Same Discrete Proportional Hazards Model as the Primary Analysis but Censoring Events Occurring After One Year Following End of Treatment
eFigure 3. Sensitivity Analysis of the Primary and Major Secondary Prostate Safety Endpoints Using the Same Discrete Proportional Hazards Model as the Primary Analysis but Censoring Events Occurring After 30 Days Following End of Treatment
eFigure 4. Changes in PSA Levels From Baseline in the TRT and Placebo Groups
eTable 1. Maximum Attained PSA Values in ng/mL Prior to Prostate Biopsy for Men Diagnosed With Prostate Cancer
eTable 2. Serum Dihydrotestosterone Concentrations

This supplementary material has been provided by the authors to give readers additional information about their work.

eAppendix 1. TRAVERSE Trial Sites and Investigators

Number of patients enrolled in parentheses

R. Helm, Investigative Clinical Research of Indiana, LLC, Elwood, IN (93); P. Trueba, Future Care Solution LLC, Miami, FL (91); S. Kester, Clinical Research Center of Florida, Pompano Beach, FL (84); S. Ansari, Longwood Research, Huntsville, AL (68); A. Makhlouf, Hillcroft Medical Clinic, Sugar Land, TX (60); V. Hoon, Advanced Biomedical Research of America Inc., Las Vegas, NV (57); S. Aslam, Northwest Houston Heart Center, Tomball, TX (51); K. Bender, DBC Research, Tamarac, FL (50); J. Cardona, Indago Research and Health Center, Inc., Hialeah, FL (47); A. Dinnerstein, Helix Biomedics, LLC, Boynton Beach, FL (47); B. Wieskopf, North Georgia Clinical Research, Woodstock, GA (47); R. Falcone, Amici Clinical Research, LLC - Raritan, Raritan, NJ (46); J. Mandry, West Orange Endocrinology, P.A., Ocoee, FL (46); E. Shapiro, Eclipse Clinical Research, Tucson, AZ (45); M. Darani, Marvel Clinical Research, LLC, Huntington Beach, CA (44); L. Pearlman, Synexus Clinical Research US, Inc. – Tempe, Chicago, IL (44); S. Nasir, Affinity Clinical Research Institute, Oak Brook, IL (43); R. Heller, Bayview Research Group LLC. Valley Village, CA (42); J. Miracle, Synexus Clinical Research US, Inc. – Tempe, Akron, OH (42); M. Digiovanna, DiGiovanna Family Care Center (DFCC), North Massapequa, NY (41); K. Lucas, Diabetes & Endocrinology Consultants, Morehead City, NC (41); N. Andrawis, Manassas Clinical Research Center, Manassas, VA (39); E. Davidson, New Phase Research & Development, LLC, Knoxville, TN (39); M. Doty, Care Research Center Inc., Miami, FL (39); D. Mishkin, InvesClinic Research Center, Fort Lauderdale, FL (38); C. Brinson, Austin Primary Care Physicians, Pflugerville, TX (37); N. Farris, The Research Group of Lexington, LLC, Lexington, KY (37); G. Kipp, Northwest Clinical Research Center, Bellevue, WA (37); A. Krishna, Diabetes and Endocrinology Associates of Stark County, Inc., Canton, OH (37); P. Moore, ClinRx Research, LLC, Plano, TX (37); G. Yeoman, Founders Research Corporation, Philadelphia, PA (37); M. Allaw, Clinical Research Advantage, Inc. Tempe, Evansville, IN (36); J. Bashour, Synexus – Dallas, Dallas, TX (35); R. Montgomery, Triad Clinical Trials, Greensboro, NC (35); A. Tan, West Coast Research, LLC, Dublin, CA (35); A. Ahmad, East Texas Cardiology, PA, Houston, TX (34); D. Levinson, Chicago Clinical Research Institute, Inc., Chicago, IL (34); G. Shockey, Desert Clinical Research, LLC, Mesa, AZ (34); K. Ayesu, Omega Research Consultants LLC, Orlando, FL (33); L. McGill, Clinical Neuroscience Solutions, Inc. (CNS Healthcare of Memphis), Memphis, TN (33); N. Secrist, Alliance for Multispecialty Research, LLC – Wichita, Wichita, KS (33); N. Gabra, Burke Internal Medicine & Research, Burke, VA (32); J. Kopp, Synexus Clinical Research US, Inc. – Tempe, Anderson, SC (32); L. McGill, Synexus Clinical Research US, Inc. / McGill Family Practice, Omaha, NE (32); N. Morrar, G & L Research, LLC, Foley, AL (32); R. Powell, Velocity Clinical Research - Cincinnati, Cincinnati, OH (32); R. Severance, East Valley Family Physicians, Chandler, AZ (32); P. Bravo, Bravo Health Care Center, North Bay Village, FL (31); R. Broughton, CB Flock Research Corporation, Mobile, AL (31); K. Cruz, Advanced Pharma CR, LLC, Miami, FL (31); S. Gorrela, Spring Family Practice Associates, PA, Spring, TX (30); B. MacGillivray, Discovery Clinical Trials, San Antonio, TX (30); J. Loy, South Florida Wellness & Clinical Research Institute, Margate, FL (29); C. Lunn, Lynn Health Science Institute East, Oklahoma City, OK (29); T. Gilford, Associates in Medicine, PA, Houston, TX (27); P. Grossman, Masters of Clinical Research, Inc., Augusta, GA (27); T. Howard, Medical Affiliated Research Center, Huntsville, AL (27); A. Mendelson, Health Awareness, Inc. – Jupiter, Jupiter, FL (27); E. Riffer, Central Phoenix Medical Clinic, LLC, Phoenix, AZ (27); C. Calinescu, Synexus - Henderson DRS, Henderson, NV (26); P. Levins, Tatum Highlands Medical Associates, Glendale, AZ (26); R. Strzinek, Protenium Clinical Research, Hurst, TX (26); F. Velazquez, Pioneer Research Solutions, Inc., Houston, TX (26); M. Billings, Synexus – Greer, Greer, SC (25); G. Debs-Perez, Harmony Clinical Research, Inc., North Miami Beach, FL (25); K. Lee, Randolph Medical Associates, Asheboro, NC (25); I. Marar, West Broadway Clinic, Council Bluffs, IA (25); R. Toma, Simon-Williamson Clinic, Birmingham, AL (25); A. Adams, AB Clinical Trials, Las Vegas, NV (24); H. Audish, Encompass Clinical Research, Spring Valley, CA (24); M. Azizad, Valley Clinical Trials, Inc., Northridge, CA (24); C. Greiwe, Synexus Clinical Research US, Inc. – Columbus, OH (24); A.L. Lewy-Alterbaum, ALL Medical Research, LLC, Cooper City, FL (24); L. Akright, Northeast Endocrinology Associates, PA, Live Oak, TX (23); A. Al-Karadsheh, The Endocrine Center, Houston, TX (23); J. DeGrauw, Wasatch Peak Family Practice, Layton, UT (23); S. Dua, Valley Renal Medical Group, Northridge, CA (23); W. Jennings, Synexus Clinical Research US, Inc. - San Antonio, San Antonio, TX (23); L. Odekirk, Lynn Institute of Denver, Aurora, CO (23); M. Trevino, Innovative Research, Clearwater, FL (23); J. Venereo, Pharmax Research Clinic, Miami, FL (23); M.

© 2023 Bhasin S et al. *JAMA Network Open.*

Agha, OnSite Clinical Solutions, LLC – Charlotte, Charlotte, NC (22); S. Benjamin, Universal Research Group, LLC, Tacoma, WA (22); M. Efros, Accumed Research Associates, Garden City, NY (22); J.C. Garza, Victorium Clinical Research, San Antonio, TX (22); V. Gold, FMC Science, LLC, Lampasas, TX (22); J. Kay, Clinical Research Advantage, Inc. - Council Bluffs, Omaha, NE (22); L. Rudolph, New Mexico Clinical Research & Osteoporosis Center, Inc., Albuquerque, NM (22); A. Fatakia, Tandem Clinical Research, LLC, Marrero, LA (21); M. Goisse, Frontier Clinical Research, LLC – Smithfield, Smithfield, PA (21); A. Hartman, Virginia Research Center, LLC, Midlothian, VA (21); K. Vora, Research Integrity, LLC, Owensboro, KY (21); A. Bhargava, Iowa Diabetes and Endocrinology Center (IDEC), West Des Moines, IA (20); L. Murray, Synexus Clinical Research US, Inc. - Pinellas Park, Pinellas Park, FL (20); T. Pluto, Frontier Clinical Research, LLC – Scottdale, Scottdale, PA (20); V. Fragoso, Texas Center for Drug Development, Inc., Houston, TX (19); S. Geller, Centennial Medical Group, Elkridge, MD (19); M. Lopez, Charlotte Heart & Vascular Institute, Port Charlotte, FL (19); R. Orr, Phoenix Medical Group, PC, Peoria, AZ (19); W. Patton, Quality of Life Medical & Research Center, Tuscon, AZ (19); B. Seidman, Seidman Clinical Trials, Delray Beach, FL (19); L. Thurman, IPS Research Company, Oklahoma City, OK (19); H. Tran, Texas Diabetes and Endocrinology - South Austin, Round Rock, TX (19); M. Winnie, Crossroads Clinical Research, LLC, Corpus Christi, TX (19); D. Allison, Hillcrest Clinics, Waco, TX (18); P. Nugent, Synexus Clinical Research US, Inc. – Cincinnati, Cincinnati, OH (18); L. Nunez, New Horizon Research Center, Miami, FL (18); M. Nunez, Synexus – Queens, Jamaica, NY (18); G. Quesada, Veritas Research, Corp., Miami Gardens, FL (18); J. Solis, Centex Studies, Inc. – McAllen, McAllen, TX (18); N. Wick, Meridien Research Inc. – Tampa, Tampa, FL (18); J. Andersen, Meridien Research, Lakeland, FL (17); W. Bowman, Sensible Healthcare, Ocoee, FL (17); D. Butuk, Solaris Clinical Research, Meridian, ID (17); L. Connery, Intend Research, Norman, OK (17); G. Freeman, Health Research of Hampton Roads, Inc. (HRHR), Newport News, VA (17); M. Janik, Accellacare US Inc. of Wilmington, Wilmington, NC (17); R. Klein, Newport Native MD, Inc., Newport Beach, CA (17); R. Lending, Synexus Clinical Research US, Inc. – Tucson, Tucson, AZ (17); T. Lenzmeier, Lenzmeier Family Medicine, Glendale, AZ (17); J. Sandberg, Oakland Medical Research – Michigan, Troy, MI (17); P. Wylie, Preferred Research Partners, Little Rock, AR (17); N. Azad, Edward Hines Jr. VA Hospital, Hines, IL (16); A. Barber, OnSite Clinical Solutions, LLC – Charlotte; Hickory, NC (16); S. Bauer, OnSite Clinical Solutions, LLC – Charlotte, Charlotte, NC (16); H. Maheshwari, Midwest Endocrinology, Crystal Lake, IL (16); F. Munoz, Union Square Medical Associates, PC, Elizabeth, NJ (16); K. Soe, Veterans Affairs North Texas Health Care System (VANTHCS), Dallas, TX (16); E. Soroka, Eugene Soroka MD, Inc., Port Hueneme, CA (16); H. Upadhyay, Sandhill Research, LLC, St. Petersburg, FL (16); J. Agaiby, Clinical Investigation Specialists, Inc., Gurnee, IL (15); C. Bird, Colorado Springs Family Practice, Colorado Springs, CO (15); G. Disick, Imagine Research of Palm Beach County, Boynton Beach, FL (15); L. Duke, DMI Research (Decision Management International Health Care Group, Inc.), Pinellas Park, FL (15); T. Gaskin, Century Clinical Research, Inc., Daytona Beach, FL (15); N. Godbole, Summit Medical Group – Glendale, Glendale, AZ (15); R. Huling, Olive Branch Family Medical Center, Olive Branch, MS (15); D. Jack, Lone Peak Family Medicine, Draper, UT (15); W. Knapp, Med Research One, Florissant, MO (15); G. Ledesma, Arlington Family Health Pavilion, Arlington, TX (15); S. Makam, Mid Hudson Medical Research, PLLC, New Windsor, NY (15); S.A. Mujica Trenche, ALAS Science Clinical Research, Henderson, NV (15); J. Schmidt, North State Clinical Research, Lenoir, NC (15); L. Tharenos, Synexus Clinical Research US, Inc. - St. Louis, St. Louis, MO (15); S. Wilson, Ocean State Clinical Research Partners, LLC, Lincoln, RI (15); R. Anderson, VA Nebraska - Western Iowa Health Care System, Omaha, NE (14); K. Barbel-Johnson, Care Partners Clinical Research, Jacksonville, FL (14); P. Barnhill, Vineland Family Medicine, Whiteville, NC (14); J. Bashour, Synexus – Plano, Plano, TX (14); D. Bouda, Heartland Clinical Research, Omaha, NE (14); J. Daniel, Clinical Research Partners, LLC - Family Medicine / Internal Medicine, Richmond, VA (14); L. Feld, Horizon Clinical Research, LLC, Gilbert, AZ (14); T. Fiel, Fiel Family & Sports Medicine, Tempe, AZ (14); S. Grubb, Waterway Primary Care, LLC - Tabor City, Little River, SC (14); M. Hummel, Fountain Hills Family Practice PC, Fountain Hills, AZ (14); S. Koch, Koch Family Medicine, Morton, IL (14); M. Kramer, MB Clinical Research, Boca Raton, FL (14); J. Pouzar, Centex Studies, Inc. – Houston, Houston, TX (14); G. Trullenque, Floridian Research Institute LLC, Miami, FL (14); M. Welch, Consano Clinical Research, Shavano Park, TX (14); E. Armas, Well Pharma Medical Research, Miami, FL (13); J. Chang, Velocity Clinical Research, North Hollywood, North Hollywood, CA (13); R. Jordan, Center for Clinical Trials of Sacramento, Inc., Sacramento, CA (13); S. Leichter, Endocrine Consultants, PC – Columbus, Columbus, GA (13); R. Perry, Panax Clinical Research, Miami Lakes, FL (13); W. Randall, PriMed Clinical Research, Dayton, OH (13); R. Sastre, APF Research, LLC, Miami, FL (13); E. Bolster,

© 2023 Bhasin S et al. *JAMA Network Open.*

Palmetto Clinical Research (PCR), Summerville, SC (12); A. Cohen, The Endocrine Clinic, PC, Memphis, TN (12); C. Griffin, Lynn Health Science Institute (LHSI), Oklahoma City, OK (12); C. Herman, Quantum Clinical Trials, Miami Beach, FL (12); J. Jacqmein, Jacksonville Center for Clinical Research, Jacksonville, FL (12); B. Khan, Atlanta Vascular Research Foundation - Atlanta Clinical Research Centers, Atlanta, GA (12); I. Lieber, Texas Cardiology Associates of Houston, Kingwood, TX (12); C. Mbogua, Discovery MM Services, Inc. - Houston - Broadway St., Houston, TX (12); A. Murcia, DBC Research USA Corporation, Pembroke Pines, FL (12); R. Patel, Endocrine and Psychiatry Center, Houston, TC (12); H. Stamps, Collierville Medical Specialists, Collierville, TN (12); R. Swerdloff, Lundquist Institute for Biomedical Innovation at Harbor - UCLA Medical Center, Torrance, CA (12); J. Whatley, Centex Studies, Inc., Lake Charles, LA (12); M. Adams, Synexus Clinical Research US, Inc. - Salt Lake City, Murray, UT (11); J. Bailen, First Urology, Jeffersonville, IN (11); M. Bidair, San Diego Clinical Trials, La Mesa, CA (11); C. Breton, International Research Associates, LLC, Miami, FL (11); N. Daboul, Advanced Medical Research, LLC – Maumee, Maumee, OH (11); G. Flippo, Alabama Clinical Therapeutics, LLC, Birmingham, AL (11); M. Franco, Memorial Clinical Associates, PA, Houston, TX (11); G. Funk, Fundamental Research, Gulf Shores, AL (11); E. Gaddam, The Loretto Hospital, Chicago, IL (11); T. Hart, Terence T. Hart MD, Tuscumbia, AL (11); D. Headley, David M. Headley, MD, PA, Port Gibson, MS (11); D. James, The University Of Tennessee Health Science Center, Memphis, TN (11); D. Koontz, Palmetto Institute of Clinical Research, Inc., Pelzer, SC (11); D. McNeil, Optimed Research, LTD, Columbus, OH (11); M. Raikhel, Torrance Clinical Research Institute, Inc., Lomita, CA (11); M. Cornett, American Health Network of IN, LLC – Franklin, Franklin, IN (10); A. Daluga, American Health Network - Family Medicine and Specialty Care Services in Greenfield, Greenfield, IN (10); R. D'Anna, Applied Research Center of Arkansas, Little Rock, AR (10); A. Gosmanov, Albany Stratton VA Medical Center, Albany, NY (10); A. Iranmanesh, Salem VA Medical Center, Salem, VA (10); A. Qureshi, Pioneer Research Solutions, Inc., Beaumont, TX (10); H. Reyes, MediSphere Medical Research Center, Evansville, IN (10); S. Rosenberg, The Iowa Clinic, PC, West Des Moines, IA (10); D. Spiller, South Florida Research Solutions, LLC, Hollywood, FL (10); R. Estevez, Clinical Research of South Nevada, Las Vegas, NV (9); B. Green, B.G. Clinical Research Center, LLC, Little Rock, AR (9); E. Hanna, Internal Medicine Associates, Bridgeton, NJ (9); R. Kastelic, Richard M. Kastelic MD & Associates, PC, Johnstown, PA (9); M. Lawrence, Carteret Medical Group - Morehead City, Morehead City, NC (9); R. Manning, PMG Research of Knoxville, Knoxville, TN (9); K. Maynard, Investigators Research Group, LLC , Indianapolis, IN (9); C. Ng, Tower Urology, Los Angeles, CA (9); L. Phillips, Atlanta VA Medical Center, Decatur, GA (9); S. Plantholt, Maryland Cardiovascular Specialists, Baltimore, MD, (9); R. Powell, Meridien Research Inc. - Spring Hill, Spring Hill, FL (9); O. Ruffin, Trinity Clinical Research, LLC, Tullahoma, TN (9); J. Walsh, Richard L. Roudebush VA Medical Center, Indianapolis, IN (9); M. Woolman, Advanced Research Institute, Ogden, UT (9); S. Arora, Aventiv Research – Columbus, Columbus, OH (8); V. Awasty, Awasty Research Network, LLC, Marion, OH (8); T. Barker, PMG Research of Piedmont Healthcare, Mooresville, NC (8); S. Butman, Verde Valley Medical Center, Tucson, AZ (8); D. Cahn, Foothills Urology, Lakewood, CO (8); S. Dhindsa, Saint Louis University School of Medicine, St. Louis, MO (8); A. Doshi, PrimeCare Medical Group, Houston, TX (8); A. Drabick, Medication Management LLC, Raleigh, NC (8); R. Hood, Discover Research, Inc., Beaumont, TX (8); M. Jardula, Desert Oasis Healthcare, Palm Springs, CA (8); J. Kovac, Urology of Indiana, Indianapolis, IN (8); D. McMullen, Discovery MM Services, Inc. - Missouri City, Missouri City, TX (8); W. Pharr, Medication Management LLC, Greensboro, NC (8); S. Phatak, Connecticut Clinical Research, LLC, Waterbury, CT (8); L. Quintero, International Research Associates, LLC – Hialeah, Hialeah, FL (8); A. Becker, Deaconess Clinic Downtown, Evansville, IN (7); B. Christine, Urology Centers of Alabama, P.C., Homewood, AL (7); W. Fitzgibbons, Skyline Medical Center, Elkhorn, NE (7); D. Fitz-Patrick, East-West Medical Research Institute, Honolulu, HI (7); N. Fraser, Troy Internal Medicine, P.C. - Research Division, Troy, MI (7); B. Gilbert, Office of Bruce R. Gilbert, MD, Great Neck, NY (7); I. Goldstein, San Diego Sexual Medicine, San Diego, CA (7); J. Haffizulla, Precision Clinical Research, LLC – Lauderdale Lakes, Lauderdale Lakes, FL (7); J. Lillo, Elite Clinical Studies, LLC, Phoenix, AZ (7); T. Nguyen, Solutions Through Advanced Research, Inc., Jacksonville, FL (7); E. Riley, Self Medical Group - Advanced Research Associates, Hodges, SC (7); D. Storey, American Health Network of IN, LLC – Avon, Avon, IN (7); S. Tebi, Care Access Research, Santa Clarita, CA (7); D. Uba, Rapha Institute for Clinical Research, Fayetteville, NC (7); J. Wayne, Clinical Trials Research, Lincoln, CA (7); D. Ajani, Discovery MM Services, Inc.– Katy, Houston, TX (6); E. Christofides, Endocrinology Research Associates, Columbus, OH (6); D. Francyk, Family Practice Specialists, Phoenix, AZ (6); D. Huffman, University Diabetes & Endocrine Consultants, Chattanooga, TN (6); S. Malempati, Tampa Bay Medical Research, Clearwater, FL (6); A.

Odugbesan, Physicians Research Associates, LLC, Lawrenceville, GA (6); B. Pierpont, Advance Medical Research, Inc., St. Petersburg, FL (6); M. Rausch, Heartland Research Associates, LLC, El Dorado, KS (6); N. Razzaque, Synexus Clinical Research US, Inc. - West Florissant Internists, Bridgeton, MO (6); K. Rictor, SFM Clinical Trials, Scotland, PA (6); J. Stewart, Family Medicine Associates of Texas, PA, Carrollton, TX (6); D. Tripathy, South Texas Veterans Health Care System - Audie L. Murphy VA Hospital, San Antonio, TX (6); T. Briskin, Velocity Clinical Research - Cleveland, Cleveland, OH (5); K. Cohen, New West Physicians, PC, Golden, CO (5); A. Dang, Facey Medical Group, Mission Hills, CA (5); K. Fox, Family Medical Associates of Highland Park, LLC, Levittown, PA (5); S. Freedman, Sheldon J. Freedman, MD, Ltd., Las Vegas, NV (5); A. George, Seven Corners Medical Center, Falls Church, VA (5); E. Goldfischer, Premier Medical Group - Urology Division, Poughkeepsie, NY (5); R. Hollister, Lynn Institute of the Rockies, Colorado Springs, CO (5); R. Jacks, Hill Country Medical Associates, New Braunfels, TX (5); K. Kelley, Biofortis Clinical Research, Inc., Addison, IL (5); R. Mills, PMG Research of Charleston, Mt Pleasant, SC (5); R. Mohseni, Catalina Research Institute, LLC, Montclair, CA (5); F. Saba, Professional Health Care of Pinellas, Inc., St. Petersburg, FL (5); J. Sensenbrenner, PMG Research of Charlotte, Charlotte, NC (5); E. Sorial, Prima CARE, P.C., Fall River, MA (5); P. Wakefield, PMG Research of Knoxville, Knoxville, TN (5); L. Whitlock, Primary Care Group, LLC, Memphis, TN (5); M. Ampajwala, ACRC Trials, Frisco, TX (4); J. Borders, Drs. Borders, Hood, and Associates, Lexington, KY (4); P. Bradley, Meridian Clinical Research, LLC – Savannah, Savannah, GA (4); O.G. Brkic-Vukotic, Atlanta Center for Clinical Research, Roswell, GA (4); A. Brockmyre, Holston Medical Group, Bristol, TN (4); J. Chehade, University of Florida Health Endocrinology – Emerson, Jacksonville, FL (4); R. Harris, DeGarmo Institute of Medical Research, Greer, SC (4); N. Jaffrani, Alexandria Cardiology Clinic, Alexandria, LA (4); J. Kaminetsky, Manhattan Medical Research, New York, NY (4); G. Ledger, Mercy Clinic Endocrinology, LLC, Springfield, MO (4); S. Lerman, The Center for Diabetes and Endocrine Care – Hollywood, Ft. Lauderdale, FL (4); A. Mabaquiao, TriWest Research Associates, El Cajon, CA (4); I.J. Madu, Diabetes Associates Medical Group, Orange, CA (4); R. Myers, "Clinic-LJ" Ltd, Ponte Vedra, FL (4); S. Nakhle, Palm Research Center, Inc., Las Vegas, NV(4); J. Perez, South Texas Cardiovascular Consultants, San Antonio, TX (4); W. Rust, Heritage Valley Medical Group, Beaver, PA (4); J. Serje, NY Total Medical Care, P.C., Brooklyn, NY (4); L. Smith, WVVA HealthCare Alliance, PC - Valley Medical Associates Inc., Lewisburg, WV (4); M. Tellez, Pacific Oaks Medical Group, Beverly Hills, CA (4); L. Belkoff, MidLantic Urology - Bala Cynwyd, Bala Cynwyd, PA (3); L. Berman, OnSite Clinical Solutions, LLC – Charlotte, Charlotte, NC (3); J. Cochran, Urology Clinics of North Texas, Dallas, TX (3); C. Cone, Montana Medical Research, Missoula, MT (3); K. Ellis, Clinical Research Associates of Tidewater, Norfolk, VA (3); S.M. Harman, Phoenix VA Health Care System, Phoenix, AZ (3); L. Herman, Herman Clinical Research, LLC, Suwanee, GA (3); J. Kirby, PMG Research of Knoxville, Jefferson City, TN (3); D. Liljenquist, Rocky Mountain Diabetes and Osteoporosis Center, Idaho Falls, ID (3); E. Morawski, Holston Medical Group, Kingsport, TN (3); N.C. Morcos, Syrentis Clinical Research, Santa Ana, CA (3); M. Patel, LaPorte County Institute for Clinical Research, Inc., Michigan City, IN (3); W. Penny, VA San Diego Healthcare System, San Diego, CA (3); E. Portnoy, Millennium Clinical Trials, Thousand Oaks, CA (3); M. Shanik, Endocrine Associates of Long Island, P.C., Smithtown, NY (3); H. Bagga, AGH Internal Medicine – Northside, Pittsburgh, PA (2); V. Bland, Bland Clinic, PA, Greensboro, NC (2); P. Bressler, North Texas Endocrine Center, Dallas, TX (2); J. Condit, American Health Network of Indiana – Muncie, Muncie, IN (2); A. Latorre, Applemed Research, Inc., Miami, FL (2); R. Leon, IMIC Inc., Palmetto Bay, FL (2); M. Marcelli, Michael E. DeBakey VA Medical Center, Houston, TX (2); M. Palatnik; Allied Clinical Research, LLC, Gold River; CA (2); B. Rizzardi, Velocity Clinical Research, Salt Lake City, West Jordan, UT (2); M. Vaughn, Cahaba Research, Inc., Birmingham, AL (2); J. Vazquez-Tanus, Research And Cardiovascular Corp., Ponce, PR (2); J. Willis, San Gabriel Clinical Research, Georgetown, TX (2); A. Alcantara, Centro de Endocrinologia Alcantara-Gonzalez, Bayamon, PR (1); K. Blaze, South Broward Research, LLC, Pembroke Pines, FL (1); W. Clark, Alaska Urological Institute, Anchorage, AK (1); G. Cortes-Maisonet, GCM Medical Group, PSC., San Juan, PR (1); T. Dixon, Tanner Clinic – Clinton, Clinton, UT (1); P. Greenberg, James J. Peters VA Medical Center, Bronx, NY (1); A. Gupta, Dayton VA Medical Center, Dayton , OH (1); C. Harper, Meridian Clinical Research, LLC – Norfolk, Norfolk, NE (1); H. Kerr, Cincinnati Veterans Affairs Medical Center, Cincinnati, OH (1); R. Leggett, Crossroads Clinical Research, LLC, Victoria, TX (1); E. Levin, VA Long Beach Healthcare System, Long Beach, CA (1); V. Mahabadi, Olive View - UCLA Medical Center, Sylmar, CA (1); B. Miranda, University of Miami - Miller School of Medicine, Miami, FL (1); J.H. Peniston, Thomas Jefferson University Hospital, Feasterville-Trevose, PA (1); A. Seftel, Cooper University Health Care, Camden, NJ (1); N. Sunkara, Optimum Clinical Research, Optimum Clinical Research, UT (1); A.

© 2023 Bhasin S et al. *JAMA Network Open.*

Wokhlu, Malcom Randall VA Medical Center, Gainesville, FL (1); B. Wolf, Allied Clinical Research – Reno, Reno, NV (1); D. Wynn, Wright Clinical Research, Alabaster, AL (1); The following sites were also initiated to begin study activities: R. Adler, Hunter Holmes McGuire VA Medical Center, Richmond, VA; A. Ahmad, Cardiovascular Association, PLLC, Humble, TX; O. Barnum, KAMP Medical Research, Inc., Natchitoches, LA; A. Clark, VA Pittsburgh Healthcare System, Pittsburgh, PA; D. Cohen-Neamie, Charter Research LLC, Winter Park, FL; A. Comulada Rivera, Advanced Clinical Research – Bayamon, Bayamon, PR; B. Cowan, Urology Associates of Colorado, Englewood, CO; M. Dawson, ACRC Trials, Austin, TX; A. Dobs, Johns Hopkins University School of Medicine, Baltimore, MD; J. Elsen, Pharmakon Inc., Evergreen Park, IL; J. Elsen, DuPage Medical Group - Downers Grove - 31st Street, Evergreen Park, IL; B. Essink, Meridian Clinical Research, LLC – Omaha, Omaha, NE; R. Farsad, Diagnamics, Encinitas, CA; B. First, Ritchken & First MDs, San Diego, CA; B. Frandsen, Sound Medical Research, Port Orchard, WA; A. Frisoli, Amici Clinical Research, LLC – Hoboken, Hoboken, NJ; N. Gabrail, Clinical Research Trials, LLC, Canton, OH; R. Garcia, Covenant Clinical Research, PA, San Antonio, TX; S. Georgeson, Medicor Cardiology, Bridgewater, NJ; J. Gilbert, St. Jude Hospital Yorba Linda, Fullerton, CA; L. Hernandez-Vazquez, Emanuelli Research and Development Center, Arecibo, PR; A. Hoffman, VA Palo Alto Health Care System, Palo Alto, CA; R. Hunter, ARA Arizona Research Associates, Tucson, AZ; D. Hurley, Family First Medical Care, LLC, Goose Creek, SC; L. Jenkins, The Ohio State University Wexner Medical Center - Department of Urology, Columbus, OH; A. Kabour, ID Clinical Research, Ltd., Toledo, OH; D. Kayne, The Medical Group of Encino, Encino, CA; Y. Kidokoro, Integrated Research Center, Inc., San Diego, CA; D. Lee, Irvine Center for Clinical Research, Irvine, CA; A. Matsumoto, VA Puget Sound Health Care System, Seattle, WA; A. Mills, Anthony Mills, MD, Inc., Los Angeles, CA; D. Mobley, Methodist Urology Associates, Houston, TX; S.P. Mokshagundam, University of Louisville, Louisville, KY; M. Pahor, University of Florida College of Medicine, Gainesville, FL; B. Purushottam, Monument Health Clinical Research, Rapid City, SD; O. Raheem, Tulane Medical Center, New Orleans, LA; S. Randhawa, S & W Clinical Research, Fort Lauderdale, FL; S. Shah, St. Joseph's Medical Associates, Inc., Stockton, CA; M. Syed, Endocrinology Associates of Armstrong – Indiana, Indiana, PA; S. Thomson, Veterans Affairs (VA) Health Care System – Tucson, Tucson, AZ; L. Torres, DeLeon Research, PLLC, Plano, TX

© 2023 Bhasin S et al. *JAMA Network Open.*

eAppendix 2. The TRAVERSE Prostate Study Committee

The Prostate Subcommittee of the TRAVERSE Study crafted the Prostate Safety Monitoring Plan and the Statistical Analyses Plan for the analyses of the prostate safety endpoints; performed the analyses of the prostate safety data, and was led by Shalender Bhasin, MB, BS, at the Harvard Medical School, Brigham and Women's Hospital in Boston, MA. The Subcommittee's members included Shalender Bhasin, MB, BS (Chair), Harvard Medical School, Brigham and Women's Hospital, Boston, MA; Ian Thompson, MD, *CHRISTUS Santa Rosa Health System and The University of Texas Health Science Center, San Antonio, TX;* Thomas G. Travison, PhD, Marcus Institute for Aging Research, Hebrew Senior Life; Division of Gerontology, Beth Israel Deaconess Medical Center, Harvard Medical School, Boston, MA; Karol M. Pencina, PhD, Harvard Medical School, Brigham and Women's Hospital in Boston, MA; Kevin A. Buhr, PhD, University of Wisconsin, Madison, WI; Kathleen Wannemuehler, PhD, University of Wisconsin, Madison, WI; Glenn R. Cunningham, MD, Baylor College of Medicine, Houston, TX; Catherine M. Tangen, Dr.Ph, University of Washington, Seattle, WA; Lauren Wilson, RNP, Brigham and Women's Hospital, Boston, MA; Neha Rupeja, Brigham and Women's Hospital, Boston, MA; Thiago Galgiano-Jucá, MD, PhD, Brigham and Women's Hospital, Boston, MA.

AbbVie Staff (ex-officio): Panagiotis Flevaris, MD, PhD (AbbVie); Sandra Fukumoto, MBA; Michael C. Snabes, MD, PhD; Anna Chan, PharmD; Elena Dubcenco, MD, MS; Xue Li, PhD; Rachel A. Preuss, MS; Samantha J. Phillips, MPE, PMP; Thomas J. Korellis, BS.

3. Funding

The trial was funded by a consortium of testosterone manufacturers led by AbbVie, Inc. (North Chicago, IL) with additional financial support provided by Endo Pharmaceuticals (Malvern, PA), Acerus Pharmaceuticals Corporation (Ontario, Canada), Upsher-Smith Laboratories, LLC (Maple Grove, MN). Dr. Bhasin was supported in part by Thje Boston Claude D. Pepper Older Americans Independence Center grant 3P3031679 from the National Institute on Aging.

4. Prostate Adjudication Committee

Michael O'Leary, MD, PAC Chair; Scott Lucia, MD, Mark A. Preston, MD; A. John Kellog Parsons, MD, MHS

5. Data Monitoring Committee

John H. Alexander, MD, MHSc (Chairman), Duke Clinical Research Institute, Duke University, Durham, NC; William Bremner, MD, PhD, University of Washington, Seattle, WA; Eric Klein, MD, Cleveland Clinic, Cleveland, OH; Darren K. McGuire, MD MHSc, University of Texas Southwestern Medical Center, Dallas, TX; Janet Wittes, PhD, Wittes LLC, Washington, D.C.; Renato D. Lopes, MD, PhD (Observer, non-voting), Duke Clinical Research Institute, Durham, NC; Andrew Armstrong, MD, ScM (ad hoc consult), Duke University Medical Center, Duke Cancer Institute Center for Prostate and Urologic Cancers, Durham, NC

© 2023 Bhasin S et al. *JAMA Network Open.*

eFigure 1. Sensitivity Analysis of the Primary and Major Secondary Prostate Safety Endpoints Using a Cox Proportional Hazards Model

	Number (%) Participants Experiencing Events	
	**TRT (N = 2596)**	**Placebo (N = 2602)**
**Primary**		
High Grade Prostate Cancer	5 (0.19%)	3 (0.12%)
**Secondary**		
Any Prostate Cancer	12 (0.46%)	11 (0.42%)
Acute Urinary Retention	20 (0.77%)	16 (0.61%)
Invasive Procedure for BPH	23 (0.89%)	12 (0.46%)
Prostate Biopsy	16 (0.62%)	14 (0.54%)
New pharmacologic treatment	101 (3.89%)	87 (3.34%)

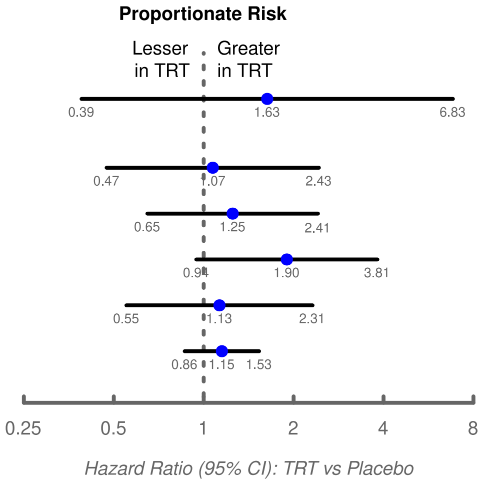

© 2023 Bhasin S et al. *JAMA Network Open.*

eFigure 2. Sensitivity Analysis of the Primary and Major Secondary Prostate Safety Endpoints Using the Same Discrete Proportional Hazards Model as the Primary Analysis but Censoring Events Occurring After One Year Following End of Treatment

	Number (%) Participants Experiencing Events	
	**TRT (N = 2596)**	**Placebo (N = 2602)**
**Primary**		
High Grade Prostate Cancer	5 (0.19%)	2 (0.08%)
**Secondary**		
Any Prostate Cancer	10 (0.39%)	9 (0.35%)
Acute Urinary Retention	18 (0.69%)	16 (0.61%)
Invasive Procedure for BPH	20 (0.77%)	11 (0.42%)
Prostate Biopsy	14 (0.54%)	12 (0.46%)
New pharmacologic treatment	89 (3.43%)	77 (2.96%)

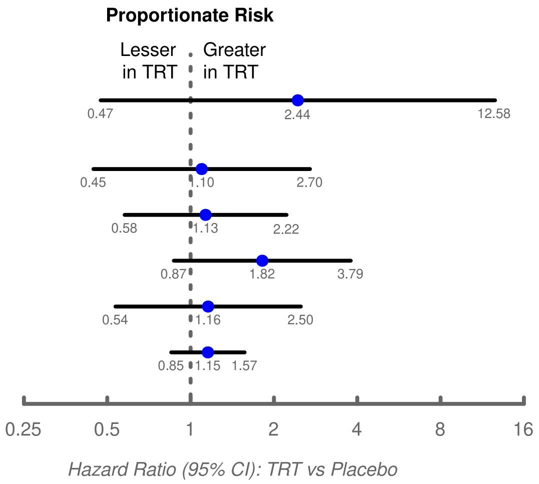

© 2023 Bhasin S et al. *JAMA Network Open.*

eFigure 3. Sensitivity Analysis of the Primary and Major Secondary Prostate Safety Endpoints Using the Same Discrete Proportional Hazards Model as the Primary Analysis but Censoring Events Occurring After 30 Days Following End of Treatment

	Number (%) Participants Experiencing Events	
	**TRT (N = 2596)**	**Placebo (N = 2602)**
**Primary**		
High Grade Prostate Cancer	3 (0.12%)	1 (0.04%)
**Secondary**		
Any Prostate Cancer	6 (0.23%)	8 (0.31%)
Acute Urinary Retention	16 (0.62%)	14 (0.54%)
Invasive Procedure for BPH	18 (0.69%)	10 (0.38%)
Prostate Biopsy	10 (0.39%)	10 (0.38%)
New pharmacologic treatment	75 (2.89%)	67 (2.57%)

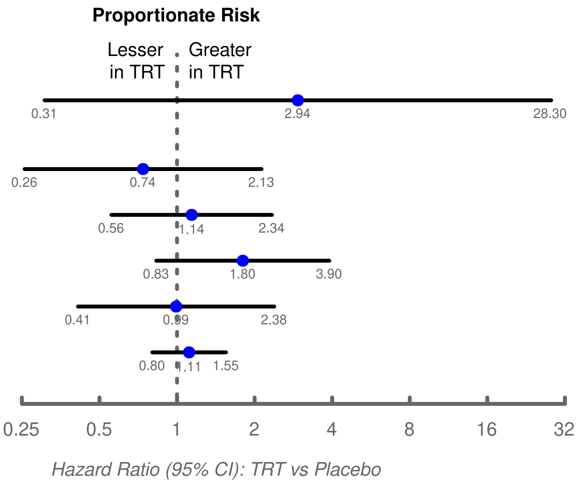

© 2023 Bhasin S et al. *JAMA Network Open.*

eFigure 4. Changes in PSA Levels From Baseline in the TRT and Placebo Groups

Symbols represent the LS mean estimates of the change from baseline, and whiskers represent the associated 95% confidence interval.

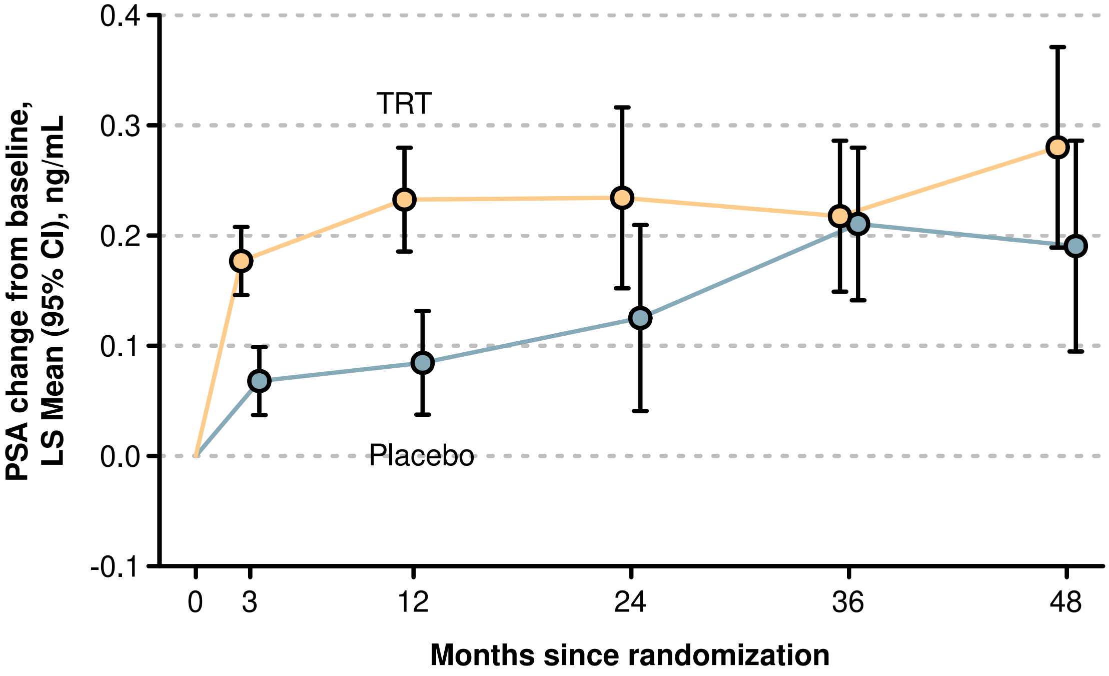

N					
Testosterone	2384	1722	1193	736	219
Placebo	2403	1729	1115	717	195

© 2023 Bhasin S et al. *JAMA Network Open.*

eTable 1. Maximum Attained PSA Values in ng/mL Prior to Prostate Biopsy for Men Diagnosed With Prostate Cancer

Subject #	Age at enrollment	Baseline PSA (ng/mL)	Maximum post-baseline PSA (ng/mL)	Prostate Cancer Grade
1	64	2.07	4.73	High
2	68	2.50	3.56	High
3	66	2.85	4.89	Low
4	61	2.85	14.4	Low
5	74	1.08	5.86	Low
6	71	2.22	4.55	Low
7	72	1.70	2.16	Low
8	63	2.40	14.76	High
9	70	2.50	3.04	Low
10	74	2.84	5.79	High
11	66	1.46	6.00	High
12	59	2.36	5.21	Low
13	68	1.70	2.35	Low
14	66	1.73	6.19	Low
15	71	2.97	4.42	High
16	51	1.23	3.69	High
17	55	2.91	8.87	Low
18	56	1.65	8.09	Low
19	66	0.83	1.38	Low
20	67	2.54	5.04	Low
21	57	3.00	5.58	Low
22	67	1.97	6.42	High
23	71	2.99	3.26	Low

Legend: High grade prostate cancer refers to cancers with Gleason score of 4+3 or higher. Low grade prostate cancers are those with Gleason score of 3+3 or 3+4.

© 2023 Bhasin S et al. *JAMA Network Open.*

eTable 2. Serum Dihydrotestosterone Concentrations

Analyte	*Month*	*Treatment*	*n*	*Value [Mean (SD)]*	*Change from Baseline*		*Treatment Difference*	
					LS Mean	*95% CI*	LS Mean	*95% CI*
Dihydrotestosterone, ng/dL	0	TRT	1768	16.1 (8.0)				
		Placebo	1754	16.4 (8.7)				
	12	TRT	1750	65.1 (51.2)	48.9	[47.2,50.6]	47.1	[44.7,49.5]
		Placebo	1735	18.2 (10.7)	1.8	[0.1,3.5]		
	36	TRT	779	68.4 (56.3)	51.7	[49.0,54.4]	48.2	[44.3,52.0]
		Placebo	768	19.9 (13.1)	3.5	[0.8,6.3]		

Legend: For each analyte, baseline counts and means (SDs) of subjects with at least one follow-up value are shown. At each post-baseline timepoint, counts and at-visit means (SDs) of subjects with data at both baseline and indicated timepoint are shown. Change from baseline for each treatment group and the difference between treatment groups (TRT minus placebo) are given as LS means with 95% confidence intervals.

To convert dihydrotestosterone (ng/dL) to SI units (nmol/L), multiply the DHT concentration by 0.0344.

Serum total testosterone and estradiol levels have been reported previously (Lincoff 2023).

© 2023 Bhasin S et al. *JAMA Network Open.*
