# Supplementary material for: Prostate Safety Events During Testosterone Replacement Therapy in Men With Hypogonadism: A Randomized Clinical Trial
Source: JAMA Netw Open. 2023 Dec 27;6(12):e2348692. doi: 10.1001/jamanetworkopen.2023.48692 (PMC10753401; doi:10.1001/jamanetworkopen.2023.48692)
Supplement: Supplement 4. — Data Sharing Statement [file jamanetwopen-e2348692-s004.pdf]

Data Sharing Statement

Bhasin. Prostate Safety Events During Testosterone Replacement Therapy in Men With Hypogonadism. *JAMA Netw Open*. Published December 27, 2023.
doi:10.1001/jamanetworkopen.2023.48692

Data
Data available: Yes
Data types: Deidentified participant data
How to access data: The data reported in this manuscript will be made available for research for noncommercial purposes upon review of a written request by the Prostate Safety Committee. The requests can be sent to sbhasin@bwh.harvard.edu
When available: beginning date: 02-01-2024

Supporting Documents
Document types: Statistical/analytic code
How to access documents: submit requests to sbhasin@bwh.harvard.edu
When available: beginning date: 02-01-2023

Additional Information
Who can access the data: researchers whose proposed use of the data has been approved by the Traverse Prostate Substudy Committee.
Types of analyses: for research purpose only
Mechanisms of data availability: after approval of a proposal, or with a signed data access agreement
Any additional restrictions: Data cannot be transferred to a third party or used for commercial purposes.
